# Dynamic and selective engrams emerge with memory consolidation

**DOI:** 10.1038/s41593-023-01551-w

**Published:** 2024-01-19

**Authors:** Douglas Feitosa Tomé, Ying Zhang, Tomomi Aida, Olivia Mosto, Yifeng Lu, Mandy Chen, Sadra Sadeh, Dheeraj S. Roy, Claudia Clopath

**Affiliations:** 1 Department of Bioengineering, Imperial College London, London, UK; 2 Institute of Science and Technology Austria, Klosterneuburg, Austria; 3 McGovern Institute for Brain Research, Massachusetts Institute of Technology, Cambridge, MA USA; 4 Center for Life Sciences & IDG/McGovern Institute for Brain Research, School of Life Sciences, Tsinghua University, Beijing, China; 5 Department of Brain Sciences, Imperial College London, London, UK; 6 Stanley Center for Psychiatric Research, Broad Institute of MIT and Harvard, Cambridge, MA USA; 7 Department of Physiology and Biophysics, Jacobs School of Medicine and Biomedical Sciences, State University of New York at Buffalo, Buffalo, NY USA

**Keywords:** Network models, Consolidation, Spike-timing-dependent plasticity

## Abstract

Episodic memories are encoded by experience-activated neuronal ensembles that remain necessary and sufficient for recall. However, the temporal evolution of memory engrams after initial encoding is unclear. In this study, we employed computational and experimental approaches to examine how the neural composition and selectivity of engrams change with memory consolidation. Our spiking neural network model yielded testable predictions: memories transition from unselective to selective as neurons drop out of and drop into engrams; inhibitory activity during recall is essential for memory selectivity; and inhibitory synaptic plasticity during memory consolidation is critical for engrams to become selective. Using activity-dependent labeling, longitudinal calcium imaging and a combination of optogenetic and chemogenetic manipulations in mouse dentate gyrus, we conducted contextual fear conditioning experiments that supported our model’s predictions. Our results reveal that memory engrams are dynamic and that changes in engram composition mediated by inhibitory plasticity are crucial for the emergence of memory selectivity.

## Abstract (web summary)

This paper shows that memory engrams are dynamic: neurons drop in and out as engrams become selective during memory consolidation. Inhibition and inhibitory plasticity are crucial for the expression and emergence of memory selectivity, respectively.

## Main

A growing body of evidence has shown that neurons activated by an experience have a prominent role in memory^1,2^. Specifically, loss-of-function and gain-of-function studies have demonstrated that ablating neurons activated during learning disrupts memory retrieval^3^, whereas artificially reactivating these neurons elicits memory recall even in the absence of retrieval cues^4^. Therefore, learning-activated neurons are a cellular substrate for memory storage and retrieval, and they constitute engram cells. The stability of engrams after memory encoding, however, remains an open question. In particular, there are two competing hypotheses regarding the effect of memory consolidation on the post-learning evolution of engrams. First, engrams may be stabilized as a result of memory consolidation (that is, stable engrams) in line with the crucial role of encoding-activated engram cells in subsequent memory retrieval^2^. Second, the relatively low overlap between ensembles of neurons activated during learning and recall (10–40%) raises the possibility that engrams may change over the course of memory consolidation with neurons ‘dropping out of’ or ‘dropping into’ the engram (that is, dynamic engrams)^2^. Critically, knowledge of the temporal profile of engrams may elucidate how engram composition is related to mnemonic properties such as memory selectivity—a feature essential for adaptive behavior^5^.

In the present study, we used a combination of computational and experimental approaches to investigate the post-encoding evolution of memory engrams. We developed a spiking neural network model that predicted that (1) neurons drop out of and drop into the engram as it switches from an unselective to a selective state over the course of memory consolidation; (2) inhibitory activity is necessary for the expression of memory selectivity during recall; and (3) inhibitory synaptic plasticity during memory consolidation is essential for the development of memory selectivity. We performed a range of contextual fear conditioning (CFC) experiments to test these predictions, and our results supported each of them. Therefore, our work demonstrated that memory engrams are dynamic and that engram cell turnover shaped by inhibitory plasticity has a crucial role in the emergence of memory selectivity. These findings challenge classical theories of stable memory traces and point to a close link between engram state and memory expression.

## Memory consolidation reshapes engrams

**Fig. 1:**
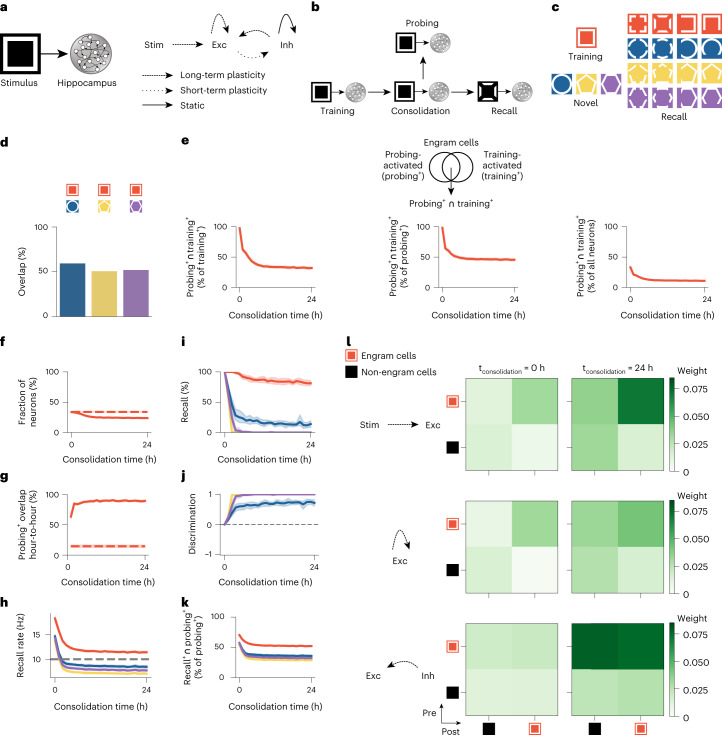
Memory consolidation renders engrams dynamic and selective. **a**, Schematic of computational model. Left, stimulus population (Stim) and hippocampus network with excitatory (Exc) and inhibitory (Inh) neurons. Right, plasticity of feedforward and recurrent synapses (Methods). **b**, Schematic of simulation protocol (Methods). **c**, Schematic of training and novel stimuli with corresponding partial cues for recall. **d**, Overlap between the training stimulus and each novel stimulus in **c** as a fraction of training stimulus neurons. **e**–**k**, Means and 99% confidence intervals are shown. *n* = 10 trials. **e**, Post-encoding evolution of engram cells. Ensemble overlap between engram cells activated during both probing and training as a fraction of training-activated engram cells (left), probing-activated engram cells (middle) and all neurons in the network (right). **f**, Ensemble of engram cells as a fraction of all neurons. Dashed line indicates engram cell ensemble at the end of training. **g**, Ensemble overlap between probing-activated engram cells at consolidation time = *t* and *t* − 1 h as a fraction of engram cells at consolidation time = *t* − 1 h. Dashed line indicates ensemble of neurons that remained part of the engram in all sampled time points (that is, consolidation time = 0, 1, …, 24 h) as a fraction of engram cells at consolidation time = 0 h (that is, training-activated engram cells). **h**, Firing rate of engram cells averaged across all cue presentations during recall as a function of consolidation time (Methods). Dashed line indicates threshold *ζ*^*thr*^ = 10 Hz for engram cell activation. Color denotes stimulus as in **c**. **i**, Memory recall as a function of consolidation time. Color denotes stimulus as in **c**. **j**, Discrimination index between recall evoked by cues of the training stimulus and individual novel stimuli as a function of consolidation time (Methods). Color denotes stimulus as in **c**. **k**, Fraction of probing-activated engram cells reactivated during recall as a function of consolidation time. Color denotes stimulus as in **c**. **l**, Mean weight strength of plastic synapses clustered according to engram cell status (that is, engram and non-engram cells). Top, feedforward excitatory synapses onto excitatory neurons. Middle, recurrent excitatory synapses onto excitatory neurons. Bottom, recurrent inhibitory synapses onto excitatory neurons. Left, at the end of the training phase. Right, after 24 h of consolidation. Representative trial is shown.

We used a computational model to probe the evolution of engram cells and their selectivity with memory consolidation. Specifically, our spiking neural network model consisted of a stimulus population that projected to the hippocampus (Fig. 1a). Feedforward and recurrent excitatory synapses onto excitatory neurons exhibited short-term and long-term plasticity, whereas inhibitory synapses onto excitatory neurons displayed inhibitory plasticity. Long-term excitatory synaptic plasticity combined a Hebbian term, consisting of triplet spike-timing-dependent plasticity (STDP)^6^, and non-Hebbian terms, including heterosynaptic plasticity^7^ and transmitter-induced plasticity^8^, as proposed previously^9^. Triplet STDP mediated Hebbian learning, and heterosynaptic plasticity prevented excessive potentiation of excitatory synapses and a consequent pathological increase in neuronal activity. Transmitter-induced plasticity prevented neurons from becoming silent. Previous work used a mean-field analysis to show that this combination of plasticity mechanisms can support stable learning and memory^9^. Inhibitory synaptic plasticity took the form of a network activity-based STDP mechanism^9^ whose primary goal was to control network activity levels^10^. Notably, inhibitory synaptic plasticity acted to ensure that the activity of the population of excitatory neurons remained at a set target level, hence subserving a homeostatic purpose (see Methods for a detailed description of the model). Our network was initially trained by presenting a training stimulus to simulate an episodic memory task (Fig. 1b,c). We then identified training-activated engram cells by examining which neurons were selectively activated in response to the training stimulus (that is, average stimulus-evoked firing rate above the threshold *ζ*^*thr*^ = 10 Hz in the last 60 s of the training phase). Subsequently, the network underwent a consolidation period when the training stimulus was reactivated in line with previous experimental reports that learning-activated sensory neurons are reactivated during post-encoding sleep^11^. At regular intervals throughout the consolidation phase (that is, consolidation time = 0, 1, …, 24 h), we investigated the state of the engram by presenting the training stimulus in a probing phase and identifying engram cells at that time point in a manner analogous to the one in the training phase. Probing-activated engram cells then represented the current state of the engram after memory consolidation. Lastly, we presented partial cues of either the training stimulus or a novel, unseen stimulus in the recall phase (Fig. 1b,c). In particular, we conducted a total of four separate recall sessions (that is, one for the training stimulus and one for each of the three novel stimuli) after every sampled consolidation interval. This allowed us to evaluate the ability of the network to selectively recall the encoded memory only when cues of the training stimulus were presented. Note that the overlap between the training stimulus (that is, square) and each novel stimulus (that is, circle, pentagon and hexagon) varies, with the circle having the highest overlap (Fig. 1d).

**Extended Data Fig. 1:**
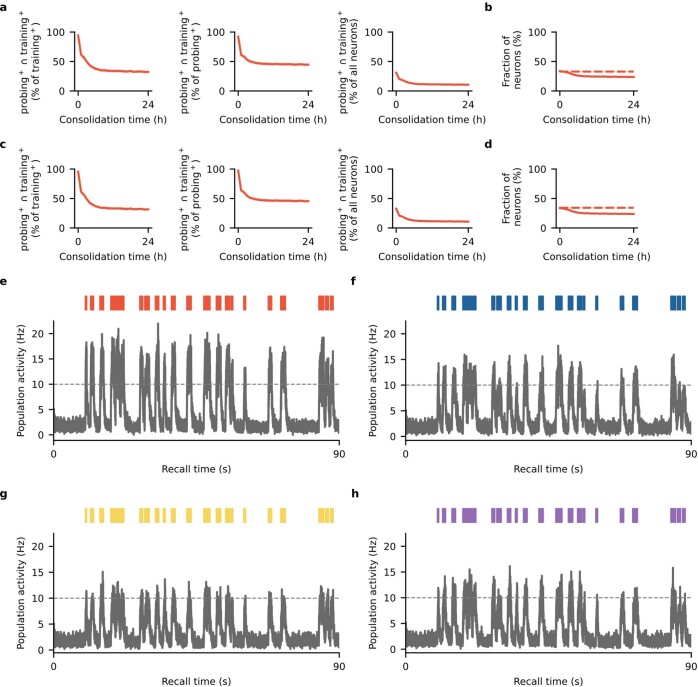
Engram cell turnover in the model is robust to the training window used to identify engram cells, and dynamic engrams are selectively reactivated by cues of the training stimulus. **a-b**, Post-encoding evolution of the engram ensemble when using the first 60 s of the training phase to identify engram cells in Fig. 1b. Means and 99% confidence intervals are shown. *n* = 10 trials. **a**, Ensemble overlap between engram cells activated during both probing and training as a fraction of training-activated engram cells (left), probing-activated engram cells (middle) and all neurons in the network (right). **b**, Engram cell ensemble as a fraction of all neurons. Dashed line indicates engram cell ensemble at the end of training. **c-d**, Post-encoding evolution of the engram ensemble when using the entire 300 s of the training phase to identify engram cells in Fig. 1b. Means and 99% confidence intervals are shown. *n* = 10 trials. **c**, Ensemble overlap between engram cells activated during both probing and training as a fraction of training-activated engram cells (left), probing-activated engram cells (middle) and all neurons in the network (right). **d**, Engram cell ensemble as a fraction of all neurons. Dashed line indicates engram cell ensemble at the end of training. **e-h**, Population activity of engram cells (that is, firing rate averaged over all engram cells) during recall after 24 h of consolidation in Fig. 1b. Population activity is shown with a temporal resolution of 10 ms without smoothing or convolution to reflect fast changes in the population response of engram cells. Note that memory recall was considered successful upon presentation of a partial cue if the firing rate of engram cells averaged over the entire cue presentation interval was above the threshold *ζ*^*thr*^ = 10 Hz for engram cell activation (see Methods). **e**, Population activity of engram cells when cues of the training stimulus were presented during recall. Cue presentation times indicated at the top where color denotes stimulus in Fig. 1c. Dashed line indicates threshold *ζ*^*thr*^ = 10 Hz for engram cell activation. Representative trial is shown. **f**, Same as **e** but when cues of a novel stimulus (that is, circle in Fig. 1c) were presented. **g**, Same as **e** but when cues of a novel stimulus (that is, pentagon in Fig. 1c) were presented. **h**, Same as **e** but when cues of a novel stimulus (that is, hexagon in Fig. 1c) were presented.

**Extended Data Fig. 2:**
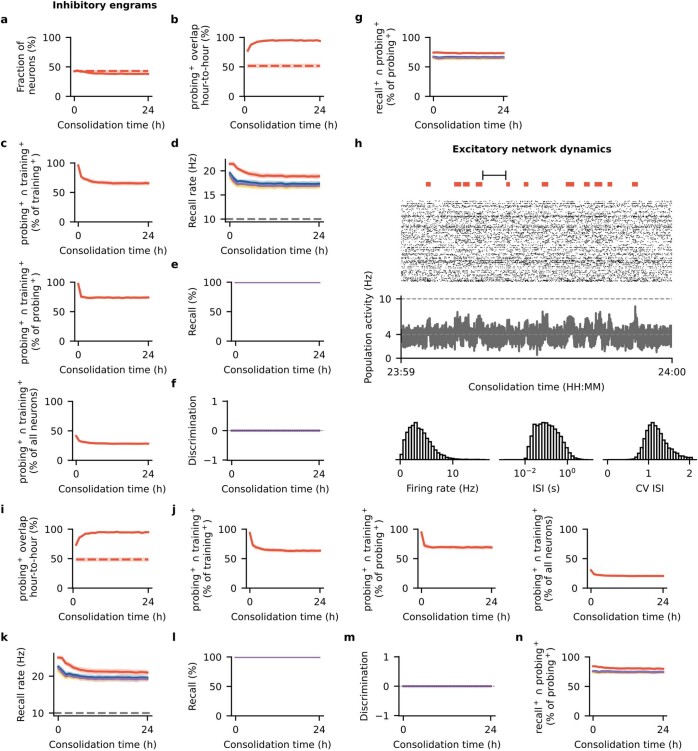
Dynamic and unselective inhibitory engrams control network activity. **a-c**, Post-encoding evolution of inhibitory engram cells in Fig. 1b. Means and 99% confidence intervals are shown. *n* = 10 trials. **a**, Ensemble of inhibitory engram cells as a fraction of all inhibitory neurons (see Methods). Dashed line indicates inhibitory engram cell ensemble at the end of training. **b**, Ensemble overlap between probing-activated inhibitory engram cells at consolidation time = *t* and *t*-1 h as a fraction of inhibitory engram cells at consolidation time = *t*-1 h. Dashed line indicates ensemble of inhibitory neurons that remained part of the inhibitory engram in all sampled time points (that is, consolidation time = 0, 1, …, 24 h) as a fraction of inhibitory engram cells at consolidation time = 0 h (that is, training-activated inhibitory engram cells). **c**, Ensemble overlap between inhibitory engram cells activated during both probing and training as a fraction of training-activated inhibitory engram cells (top), probing-activated inhibitory engram cells (middle) and all inhibitory neurons in the network (bottom). **d-g**, Analysis of inhibitory engram cells during recall in Fig. 1b. Means and 99% confidence intervals are shown. *n* = 10 trials. Color denotes stimulus as in Fig. 1c. **d**, Recall rate of inhibitory engram cells as a function of consolidation time. Dashed line indicates threshold *ζ*^*thr*^ = 10 Hz for engram cell activation. **e**, Recall of inhibitory engram cells as a function of consolidation time. **f**, Discrimination index between recall of inhibitory engram cells evoked by cues of the training stimulus and individual novel stimuli as a function of consolidation time. **g**, Fraction of probing-activated inhibitory engram cells reactivated during recall as a function of consolidation time. **h**, Analysis of excitatory network activity in a 60 s interval during consolidation in Fig. 1b. From top to bottom: training stimulus reactivation times (in red), spike raster of 256 randomly-chosen excitatory neurons (for clarity, only every fifth spike is shown), population activity (that is, average firing rate) of all excitatory neurons (dashed lines indicate target activity level *γ* = 4 Hz and threshold *ζ*^*thr*^ = 10 Hz for engram cell activation) and network statistics for the interval marked at the top (from left to right: histograms of firing rates, interspike intervals (ISI) and coefficient of variation (CV) of ISI). Population activity is shown without smoothing or convolution (see Methods). Representative trial is shown. **i-n**, Analysis of inhibitory engram dynamics in Fig. 1b using non-negative matrix factorization to identify engram cells (see Methods). Means and 99% confidence intervals are shown. *n* = 10 trials. **i**, Ensemble overlap between probing-activated inhibitory engram cells at consolidation time = *t* and *t*-1 h as a fraction of inhibitory engram cells at consolidation time = *t*-1 h. Dashed line indicates ensemble of inhibitory neurons that remained part of the inhibitory engram in all sampled time points (that is, consolidation time = 0, 1, …, 24 h) as a fraction of inhibitory engram cells at consolidation time = 0 h (that is, training-activated inhibitory engram cells). **j**, Ensemble overlap between inhibitory engram cells activated during both probing and training as a fraction of training-activated inhibitory engram cells (left), probing-activated inhibitory engram cells (middle) and all inhibitory neurons in the network (right). **k-n**, Color denotes stimulus as in Fig. 1c. **k**, Recall rate of inhibitory engram cells as a function of consolidation time. Dashed line indicates threshold *ζ*^*thr*^ = 10 Hz for engram cell activation. **l**, Recall of inhibitory engram cells as a function of consolidation time. **m**, Discrimination index between recall of inhibitory engram cells evoked by cues of the training stimulus and individual novel stimuli as a function of consolidation time. **n**, Fraction of probing-activated inhibitory engram cells reactivated during recall as a function of consolidation time.

Our simulation results showed that memory consolidation reorganized engrams with neurons being removed from and added to the engram (Fig. 1e). We tracked the fraction of training-activated engram cells that remained part of the engram over the course of memory consolidation by computing the ensemble overlap between probing-activated engram cells and training-activated engram cells as a fraction of training-activated engram cells. Given that this ratio decreased substantially as consolidation progressed, our model predicted that a large fraction of training-activated engram cells are removed from the engram and no longer actively encode the memory acquired during training (that is, they stop being activated by the training stimulus). To determine what fraction of probing-activated engram cells were originally training-activated engram cells, we computed the ensemble overlap between probing-activated engram cells and training-activated engram cells as a fraction of probing-activated engram cells. This quantity also decreased substantially with memory consolidation. Thus, our model predicted that neurons that were not activated during training are recruited into the engram and start actively encoding the underlying memory (that is, they become responsive to the training stimulus). Additionally, we found that the ensemble overlap between probing-activated engram cells and training-activated engram cells as a fraction of all neurons in the network slowly decreased over the course of consolidation. This was consistent with the ensemble of probing-activated engram cells gradually shrinking (Fig. 1f). Furthermore, our network exhibited a high overlap between probing-activated engram cell ensembles identified in two consecutive hours, but only a small fraction of training-activated engram cells remained part of the engram in every sampled consolidation interval (Fig. 1g). Note that using the first 60 s or the entire 300 s of the training phase to identify engram cells yielded analogous engram dynamics (compare Extended Data Fig. 1a–d to Fig. 1e,f). Therefore, our model predicted that memory engrams are highly dynamic, with neurons being removed from and added to the engram over the course of memory consolidation. Dynamic inhibitory engrams also emerged in our network model but with a more moderate turnover rate (Extended Data Fig. 2a–c).

In addition, engrams in our model were initially unselective but became selective over the course of memory consolidation (Fig. 1h–j). To examine engram selectivity, we first defined recall rate as the cue-evoked population firing rate of engram cells averaged across all cue presentations in the recall phase (Methods). We saw that the recall rate elicited by cues of the training and novel stimuli were above the threshold *ζ*^*thr*^ = 10 Hz for engram cell activation immediately after training, but, as memory consolidation progressed, only the recall rate of cues of the training stimulus remained above the activation threshold (Fig. 1h). We also specifically measured memory recall by computing the fraction of cue presentations in the recall phase that activated the engram cell ensemble (an engram cell ensemble was considered activated when its average firing rate was above the activation threshold during the presentation of a cue; Methods). This recall metric revealed equal memory recall levels for the training and novel stimuli immediately after training, but, with memory consolidation, only the recall of the training stimulus remained at a high level (Fig. 1i and Extended Data Fig. 1e–h). We then defined a discrimination index for memory recall as the difference between recall of the training stimulus and recall of a novel stimulus divided by their sum to provide a normalized measure of memory selectivity (Methods). This discrimination index showed that our network developed the ability to distinguish between the training and novel stimuli as a result of memory consolidation (Fig. 1j). The slightly lower post-consolidation discrimination index of one of the novel stimuli (that is, circle) can be attributed to its higher overlap with the training stimulus as mentioned previously (Fig. 1d). Thus, our model predicted that engrams are initially encoded in an unselective state but later become selective as their composition changes with memory consolidation. This is consistent with a recent study showing that memory selectivity in conditioned taste aversion emerges in the timescale of hours^12^. Note, also, that even though the fraction of probing-activated engram cells reactivated during recall was higher for the training stimulus relative to the novel stimuli, this ratio was only approximately 50% for the training stimulus after memory consolidation (Fig. 1k). This suggested that our network model performed an operation akin to pattern separation—a process that has been ascribed to the dentate gyrus (DG) and CA3 of the hippocampus^13^. Lastly, inhibitory engrams remained unselective in our simulations (Extended Data Fig. 2d–g).

The changes in engram composition and selectivity observed in our model were associated with ongoing synaptic plasticity during memory consolidation (Fig. 1l). Feedforward synapses from training stimulus neurons (that is, sensory engram cells; Methods) onto hippocampal engram cells were strengthened over the course of memory consolidation, and, consequently, the synaptic coupling between the stimulus population and the hippocampus network was increased. Recurrent excitatory synapses between engram cells also experienced a modest gain in synaptic efficacy. Notably, inhibitory synapses from inhibitory engram cells onto both engram and non-engram cells were strongly potentiated throughout memory consolidation. This indicated that a number of training-activated engram cells were forced out of the engram due to strong inhibition, and, consequently, only neurons highly responsive to the training stimulus remained in the engram, in line with our previous analysis (Fig. 1e). Inhibitory neurons also controlled the overall activity of excitatory neurons in the network through inhibitory synaptic plasticity (Extended Data Fig. 2h).

**Extended Data Fig. 3:**
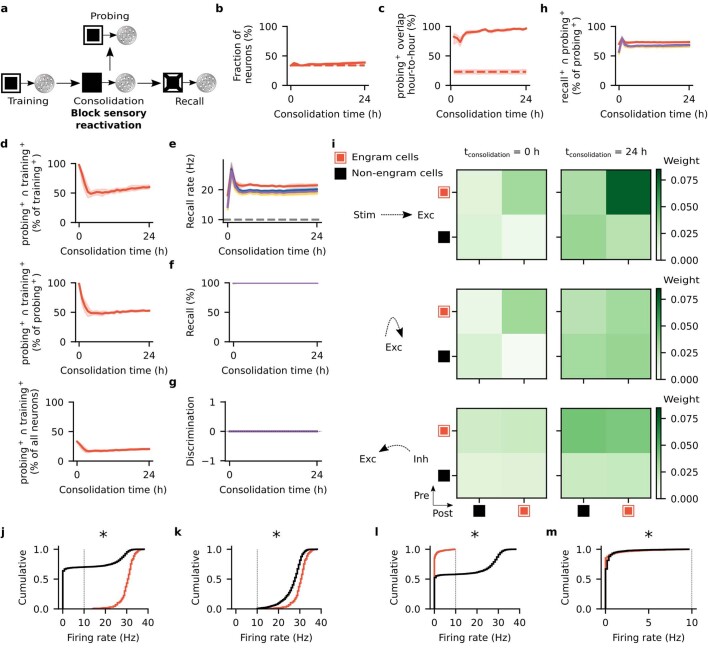
Sensory reactivation during memory consolidation is essential for engram selectivity, and analysis of stimulus-evoked neuronal firing rates during training. **a-i**, Simulation with blockage of training-activated stimulus neurons during consolidation. **a**, Schematic of simulation protocol with blockage of training-activated stimulus neurons during consolidation. Network and simulation parameters as in Fig. 1b. **b-d**, Post-encoding evolution of engram cells in **a**. Means and 99% confidence intervals are shown. *n* = 10 trials. **b**, Engram cell ensemble as a fraction of all neurons (see Methods). Dashed line indicates engram cell ensemble at the end of training. **c**, Ensemble overlap between probing-activated engram cells at consolidation time = *t* and *t*-1 h as a fraction of engram cells at consolidation time = *t*-1 h. Dashed line indicates ensemble of neurons that remained part of the engram in all sampled time points (that is, consolidation time = 0, 1, …, 24 h) as a fraction of engram cells at consolidation time = 0 h (that is, training-activated engram cells). **d**, Ensemble overlap between engram cells activated during both probing and training as a fraction of training-activated engram cells (top), probing-activated engram cells (middle) and all neurons in the network (bottom). **e-h**, Analysis of memory recall in **a**. Means and 99% confidence intervals are shown. *n* = 10 trials. Color denotes stimulus as in Fig. 1c. **e**, Firing rate of engram cells averaged across all cue presentations during recall as a function of consolidation time. Dashed line indicates threshold *ζ*^*thr*^ = 10 Hz for engram cell activation. **f**, Memory recall as a function of consolidation time. **g**, Discrimination index between recall evoked by cues of the training stimulus and individual novel stimuli as a function of consolidation time (see Methods). **h**, Fraction of probing-activated engram cells reactivated during recall as a function of consolidation time. **i**, Mean weight strength of plastic synapses in the network in **a** clustered according to engram cell status. Top, feedforward excitatory synapses onto excitatory neurons. Middle, recurrent excitatory synapses onto excitatory neurons. Bottom, recurrent inhibitory synapses onto excitatory neurons. Left, at the end of the training phase. Right, after 24 h of consolidation. Representative trial is shown. **j-m**, Analysis of the distribution of stimulus-evoked neuronal firing rates in the training phase in Fig. 1b (dashed line indicates threshold *ζ*^*thr*^ = 10 Hz for engram cell activation). **j**, Cumulative distributions of stimulus-evoked neuronal firing rates in the training phase in Fig. 1b. Red, training-activated engram cells that remained part of the engram in all sampled time points (that is, consolidation time = 0, 1, …, 24 h). Black, all other neurons. Two-sided Kolmogorov-Smirnov test, *P* = 1.776357 × 10^−15^. **k**, Cumulative distributions of stimulus-evoked neuronal firing rates in the training phase in Fig. 1b. Red, training-activated engram cells that remained part of the engram in all sampled time points (that is, consolidation time = 0, 1, …, 24 h). Black, training-activated engram cells that dropped out of the engram in any of the sampled time points (that is, consolidation time = 0, 1, …, 24 h). Two-sided Kolmogorov-Smirnov test, *P* = 3.330669 × 10^−16^. **l**, Cumulative distributions of stimulus-evoked neuronal firing rates in the training phase in Fig. 1b. Red, neurons that were not engram cells at the end of training but dropped into the engram in any of the sampled time points (that is, consolidation time = 0, 1, …, 24 h). Black, all other neurons. Two-sided Kolmogorov-Smirnov test, *P* = 0.0. **m**, Cumulative distributions of stimulus-evoked neuronal firing rates in the training phase in Fig. 1b. Red, neurons that were not engram cells at the end of training but dropped into the engram in any of the sampled time points (that is, consolidation time = 0, 1, …, 24 h). Black, neurons that were not engram cells at the end of training and remained non-engram cells in all sampled time points (that is, consolidation time = 0, 1, …, 24 h). Two-sided Kolmogorov-Smirnov test, *P* = 1.060787 × 10^−40^. **j–m**, *P < 0.05.

**Extended Data Fig. 4:**
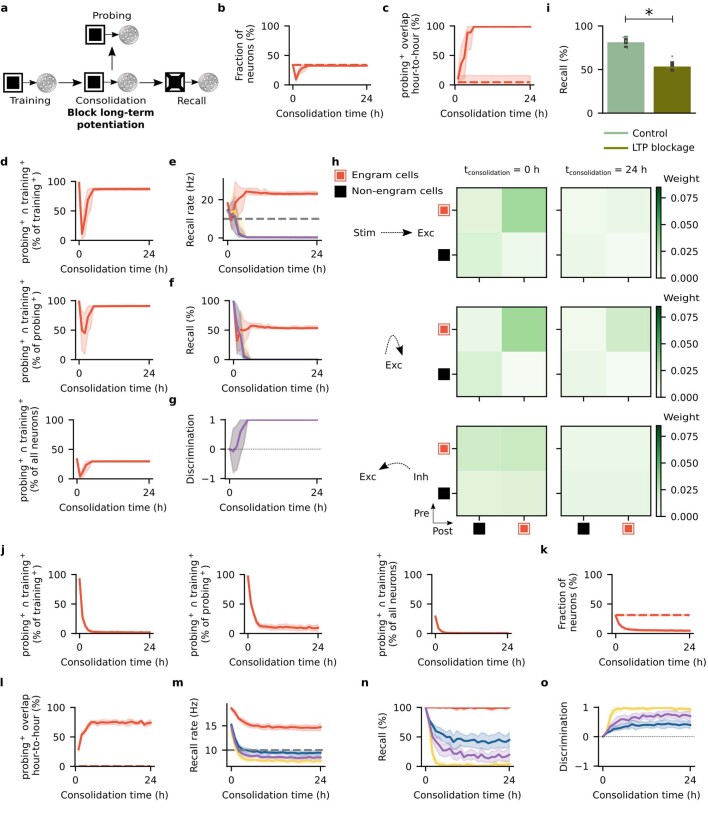
Blocking long-term potentiation during memory consolidation impairs recall, and neuronal population-based analysis yields dynamic and selective engrams. **a-i**, Simulation with blockage of long-term potentiation (LTP) during consolidation. **a**, Schematic of simulation protocol with blockage of long-term potentiation (LTP) during consolidation. LTP induced by triplet STDP and transmitter-induced plasticity are both blocked. Network and simulation parameters as in Fig. 1b except that *A* = *δ* = 0 during consolidation (see Methods). **b-d**, Post-encoding evolution of engram cells in **a**. Means and 99% confidence intervals are shown. *n* = 10 trials. **b**, Engram cell ensemble as a fraction of all neurons. Dashed line indicates engram cell ensemble at the end of training. **c**, Ensemble overlap between probing-activated engram cells at consolidation time = *t* and *t*-1 h as a fraction of engram cells at consolidation time = *t*-1 h. Dashed line indicates ensemble of neurons that remained part of the engram in all sampled time points (that is, consolidation time = 0, 1, …, 24 h) as a fraction of engram cells at consolidation time = 0 h (that is, training-activated engram cells). **d**, Ensemble overlap between engram cells activated during both probing and training as a fraction of training-activated engram cells (top), probing-activated engram cells (middle) and all neurons in the network (bottom). **e-g**, Analysis of memory recall in **a**. Means and 99% confidence intervals are shown. *n* = 10 trials. Color denotes stimulus as in Fig. 1c. **e**, Firing rate of engram cells averaged across all cue presentations during recall as a function of consolidation time. Dashed line indicates threshold *ζ*^*thr*^ = 10 Hz for engram cell activation. **f**, Memory recall as a function of consolidation time. **g**, Discrimination index between recall evoked by cues of the training stimulus and individual novel stimuli as a function of consolidation time. **h**, Mean weight strength of plastic synapses in the network in **a** clustered according to engram cell status. Top, feedforward excitatory synapses onto excitatory neurons. Middle, recurrent excitatory synapses onto excitatory neurons. Bottom, recurrent inhibitory synapses onto excitatory neurons. Left, at the end of the training phase. Right, after 24 h of consolidation. Representative trial is shown. **i**, Memory recall evoked by cues of the training stimulus at t_consolidation_ = 24 h in Fig. 1i (control) and in **f** (LTP blockage) (same data). Two-sided Wilcoxon signed-rank test, *W* = 0.0, *P* = 0.001953. Means and standard deviations are shown. *n* = 10 trials per group. *P < 0.05. **j-o**, Analysis of engram dynamics in Fig. 1b using non-negative matrix factorization to identify engram cells (see Methods). Means and 99% confidence intervals are shown. *n* = 10 trials. **j**, Post-encoding evolution of engram cells. Ensemble overlap between engram cells activated during both probing and training as a fraction of training-activated engram cells (left), probing-activated engram cells (middle) and all neurons in the network (right). **k**, Ensemble of engram cells as a fraction of all neurons. Dashed line indicates engram cell ensemble at the end of training. **l**, Ensemble overlap between probing-activated engram cells at consolidation time = *t* and *t*-1 h as a fraction of engram cells at consolidation time = *t*-1 h. Dashed line indicates ensemble of neurons that remained part of the engram in all sampled time points (that is, consolidation time = 0, 1, …, 24 h) as a fraction of engram cells at consolidation time = 0 h (that is, training-activated engram cells). Note that the dashed line is close to 0. **m-o**, Color denotes stimulus as in Fig. 1c. **m**, Firing rate of engram cells averaged across all cue presentations during recall as a function of consolidation time. Dashed line indicates threshold *ζ*^*thr*^ = 10 Hz for engram cell activation. **n**, Memory recall as a function of consolidation time. **o**, Discrimination index between recall evoked by cues of the training stimulus and individual novel stimuli as a function of consolidation time.

**Extended Data Fig. 5:**
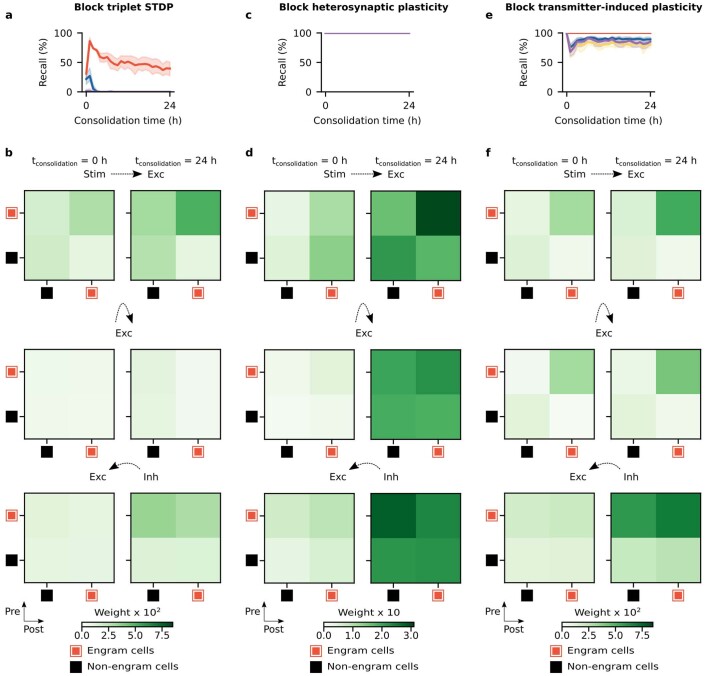
Hebbian and non-Hebbian forms of excitatory synaptic plasticity are critical for memory encoding and consolidation. **a-b**, Simulation protocol as in Fig. 1b but with blocked triplet STDP (that is, *A* = *B*_*i*_(*t*) = 0, see Methods). **a**, Memory recall as a function of consolidation time. Means and 99% confidence intervals are shown. *n* = 10 trials. Color denotes stimulus as in Fig. 1c. **b**, Mean weight strength of plastic synapses clustered according to engram cell status. Top, feedforward excitatory synapses onto excitatory neurons. Middle, recurrent excitatory synapses onto excitatory neurons. Bottom, recurrent inhibitory synapses onto excitatory neurons. Left, at the end of the training phase. Right, after 24 h of consolidation. Representative trial is shown. **c-d**, Same as **a-b** but with blocked heterosynaptic plasticity (that is, *β* = 0, see Methods). **e-f**, Same as **a-b** but with blocked transmitter-induced plasticity (that is, *δ* = 0, see Methods).

**Extended Data Fig. 6:**
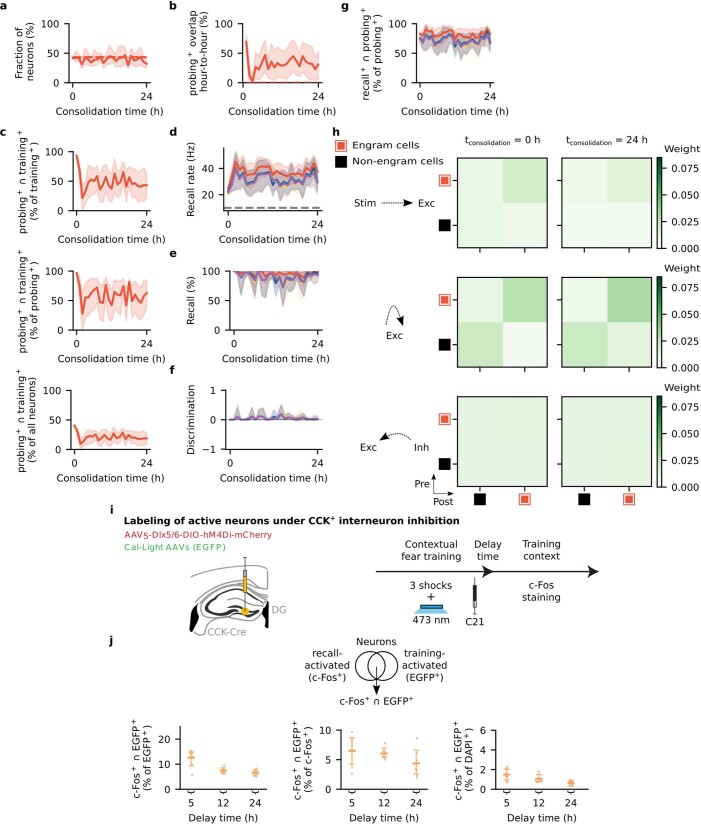
Inhibitory synaptic plasticity is essential for the emergence of memory selectivity, and engram cell turnover under CCK^+^ interneuron inhibition. **a-h**, Simulation protocol as in Fig. 1b but without inhibitory synaptic plasticity in the entire simulation. **a-g**, Means and 99% confidence intervals are shown. *n* = 10 trials. **a**, Ensemble of engram cells as a fraction of all neurons (see Methods). Dashed line indicates engram cell ensemble at the end of training. **b**, Ensemble overlap between probing-activated engram cells at consolidation time = *t* and *t*-1 h as a fraction of engram cells at consolidation time = *t*-1 h. Dashed line indicates ensemble of neurons that remained part of the engram in all sampled time points (that is, consolidation time = 0, 1, …, 24 h) as a fraction of engram cells at consolidation time = 0 h (that is, training-activated engram cells). Note that the dashed line is close to 0. **c**, Ensemble overlap between engram cells activated during both probing and training as a fraction of training-activated engram cells (top), probing-activated engram cells (middle) and all neurons in the network (bottom). **d-g**, Color denotes stimulus as in Fig. 1c. **d**, Firing rate of engram cells averaged across all cue presentations during recall as a function of consolidation time. Dashed line indicates threshold *ζ*^*thr*^ = 10 Hz for engram cell activation. **e**, Memory recall as a function of consolidation time. **f**, Discrimination index between recall evoked by cues of the training stimulus and individual novel stimuli as a function of consolidation time. **g**, Fraction of probing-activated engram cells reactivated during recall as a function of consolidation time. **h**, Mean weight strength of synapses clustered according to engram cell status. Top, feedforward excitatory synapses onto excitatory neurons. Middle, recurrent excitatory synapses onto excitatory neurons. Bottom, recurrent inhibitory synapses onto excitatory neurons. Left, at the end of the training phase. Right, after 24 h of consolidation. Representative trial is shown. **i**, Schematic of experimental protocol to track the evolution of the fear memory engram under CCK^+^ interneuron inhibition. Cal-Light was initially used to label training-activated DG neurons during CFC in mice. DG CCK^+^ interneurons were then chemogenetically inhibited immediately after fear training using C21. Following a post-training delay period, mice were placed in the training context and *c*-*Fos* staining was used to label recall-activated DG neurons. **j**, Ensemble overlap between neurons activated during both recall and training (*c-Fos*^+^ ∩ EGFP^+^) in **i** as a fraction of training-activated neurons (EGFP^+^) (left), recall-activated neurons (*c-Fos*^+^) (middle) and all cell counts (DAPI^+^) (right). These ensemble overlap ratios decreased over time, thus indicating that neurons dropped out of and dropped into the engram over the course of memory consolidation. Means and 99% confidence intervals are shown. *n* = 7 mice per group. Source data

**Extended Data Fig. 7:**
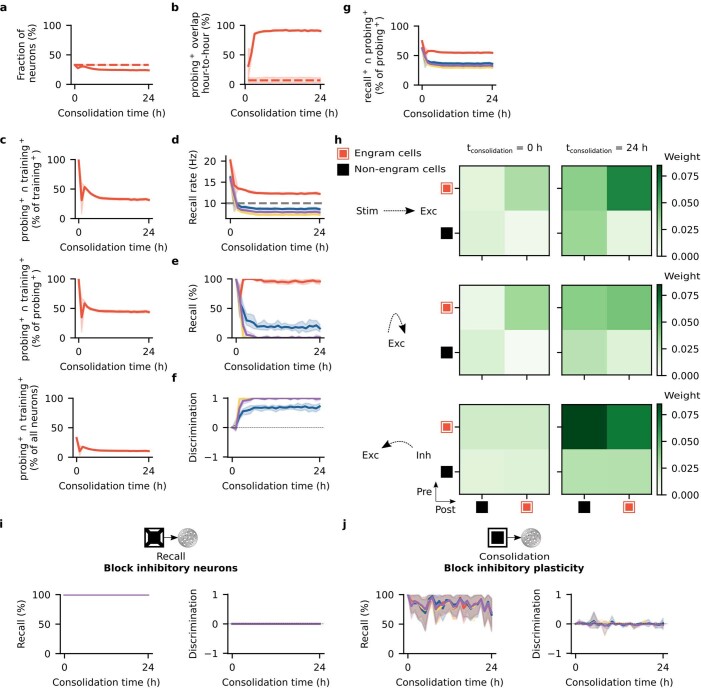
Alternative form of inhibitory synaptic plasticity supports dynamic and selective engrams. **a-h**, Simulation protocol as in Fig. 1b but with the network subjected to an alternative form of inhibitory synaptic plasticity (Equation 18, see Methods). **a-g**, Means and 99% confidence intervals are shown. *n* = 10 trials. **a**, Engram cell ensemble as a fraction of all neurons (see Methods). Dashed line indicates engram cell ensemble at the end of training. **b**, Ensemble overlap between probing-activated engram cells at consolidation time = *t* and *t*-1 h as a fraction of engram cells at consolidation time = *t*-1 h. Dashed line indicates ensemble of neurons that remained part of the engram in all sampled time points (that is, consolidation time = 0, 1, …, 24 h) as a fraction of engram cells at consolidation time = 0 h (that is, training-activated engram cells). **c**, Ensemble overlap between engram cells activated during both probing and training as a fraction of training-activated engram cells (top), probing-activated engram cells (middle) and all neurons in the network (bottom). **d**, Firing rate of engram cells averaged across all cue presentations during recall as a function of consolidation time. Dashed line indicates threshold *ζ*^*thr*^ = 10 Hz for engram cell activation. **e**, Memory recall as a function of consolidation time. **f**, Discrimination index between recall evoked by cues of the training stimulus and individual novel stimuli as a function of consolidation time. **g**, Fraction of probing-activated engram cells reactivated during recall as a function of consolidation time. **h**, Mean weight strength of plastic synapses clustered according to engram cell status. Top, feedforward excitatory synapses onto excitatory neurons. Middle, recurrent excitatory synapses onto excitatory neurons. Bottom, recurrent inhibitory synapses onto excitatory neurons. Left, at the end of the training phase. Right, after 24 h of consolidation. Representative trial is shown. **i-j**, Means and 99% confidence intervals are shown. *n* = 10 trials. **i**, Simulation protocol as in Fig. 5a but with the network subjected to an alternative form of inhibitory synaptic plasticity as in **a-h** (see Methods). Left, memory recall as a function of consolidation time. Right, discrimination index between recall evoked by cues of the training stimulus and individual novel stimuli as a function of consolidation time. **j**, Simulation protocol as in Fig. 6a but with the network subjected to an alternative form of inhibitory synaptic plasticity as in **a-h** (see Methods). Left, memory recall as a function of consolidation time. Right, discrimination index between recall evoked by cues of the training stimulus and individual novel stimuli as a function of consolidation time. **d-g/i-j**, Color denotes stimulus as in Fig. 1c. **a-j**, Network simulation parameters as in Fig. 1a-c except that ${T}_{Off}^{consolidation}=2.5$ s (see Methods and Supplementary Table 2).

To investigate the contribution of synaptic plasticity to the engram dynamics in our model, we performed several manipulations in our simulations. First, we blocked the reactivation of the training stimulus during memory consolidation and found that this altered the temporal profile of engrams and prevented them from becoming selective (Extended Data Fig. 3a–i). These effects were associated with reduced potentiation of inhibitory synapses onto engram cells (compare Extended Data Fig. 3i to Fig. 1l, bottom rows). Previous experiments demonstrated that sleep-specific inhibition of learning-activated sensory neurons disrupts memory selectivity^11^, and, hence, our model was consistent with these findings, and it predicted underlying mechanisms. Second, blocking long-term potentiation (LTP) during memory consolidation almost completely eliminated engram cell turnover after a steady state was reached, and it also impaired memory recall relative to the control case (Extended Data Fig. 4a–i). Reduced feedforward and recurrent excitatory synaptic weights due to LTP blockage led to engram stabilization and impaired recall (compare Extended Data Fig. 4h to Fig. 1l, top and middle rows). These results are in line with a recent study showing that memory recall is impaired when LTP is optically erased selectively during sleep^14^. Third, we separately blocked the Hebbian and non-Hebbian forms of long-term excitatory synaptic plasticity in our model and verified that each was essential for memory encoding and consolidation (Extended Data Fig. 5). These results are consistent with a previously reported mean-field analysis showing that this combination of plasticity mechanisms can support stable memory formation and recall^9^. Fourth, we blocked inhibitory synaptic plasticity in our entire simulation protocol, and this disrupted the emergence of memory selectivity in our network model (Extended Data Fig. 6a–h). This demonstrated that excitatory synaptic plasticity alone could not drive an increase in memory selectivity because it could not increase competition among excitatory neurons in the absence of inhibitory synaptic plasticity (compare Extended Data Fig. 6h to Fig. 1l). However, excitatory synaptic plasticity could promote engram cell turnover on its own in an even more pronounced manner than in the presence of both excitatory and inhibitory synaptic plasticity (compare Extended Data Fig. 6b to Fig. 1g). Finally, we found that an alternative inhibitory synaptic plasticity formulation yielded engram dynamics analogous to those in our original network (compare Extended Data Fig. 7a–h to Fig. 1e–l). This suggested that the dynamic and selective engrams predicted by our model are not a product of a specific form of inhibitory plasticity but a consequence of memory encoding and consolidation in inhibition-stabilized plastic networks in general.

**Fig. 2:**
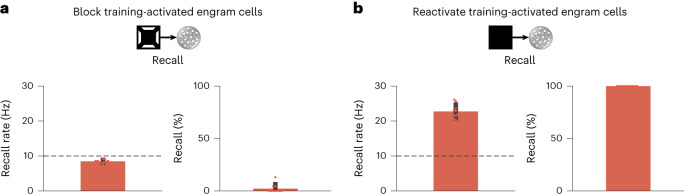
Training-activated engram cells remain necessary and sufficient for memory recall. Analysis of memory recall when training-activated engram cells in Fig. 1b are manipulated. Means and standard deviations are shown. *n* = 10 trials. **a**, Analysis of memory recall after 24 h of consolidation when training-activated engram cells are blocked during recall with cues of the training stimulus in the protocol in Fig. 1b. Left, firing rate of engram cells averaged across all cue presentations during recall (dashed line indicates threshold *ζ*^*thr*^ = 10 Hz for engram cell activation). Right, memory recall. **b**, Analysis of memory recall after 24 h of consolidation when training-activated engram cells are artificially reactivated during recall without any stimulus cues in the protocol in Fig. 1b. Left, firing rate of engram cells averaged across all reactivation intervals during recall (dashed line indicates threshold *ζ*^*thr*^ = 10 Hz for engram cell activation). Right, memory recall (note that standard deviation = 0 as recall = 100% in all trials).

We also conduced loss-of-function and gain-of-function manipulations to examine the role of training-activated engram cells in memory recall in our model (Fig. 2). We found that blocking training-activated engram cells after a consolidation period of 24 h prevented memory recall (Fig. 2a), whereas artificially reactivating them in the absence of retrieval cues was able to elicit recall (Fig. 2b), in a manner consistent with previous experimental findings^3,4^ and despite the dynamic nature of engrams in our simulations (Fig. 1e–g). Thus, our model was able to reconcile the prominent role of training-activated engram cells in memory storage and retrieval with dynamic memory engrams. To determine whether neuronal activity during memory acquisition was predictive of neurons dropping out of or dropping into the engram, we examined the distribution of stimulus-evoked neuronal firing rates in the training phase (Extended Data Fig. 3j–m). We found that training-activated engram cells that remained part of the engram throughout memory consolidation exhibited higher stimulus-evoked firing rates than (1) the remaining neurons in the network (Extended Data Fig. 3j) and (2) training-activated engram cells that dropped out of the engram over the course of consolidation (Extended Data Fig. 3k). Therefore, stimulus-evoked firing rates during training were indicative of a neuron’s ability to outlast inhibition and remain part of the engram after initial memory encoding. We also verified that neurons that were not engram cells at the end of training but later dropped into the engram displayed lower training stimulus-evoked firing rates than the remaining neurons in the network (Extended Data Fig. 3l). Surprisingly, neurons that dropped into the engram after training showed slightly lower stimulus-evoked firing rates than neurons that failed to become part of the engram altogether (Extended Data Fig. 3m). This suggested that stimulus-evoked firing rates during memory acquisition may not be reliable predictors of a neuron’s ability to increase its response to the training stimulus and become an engram cell after encoding. Lastly, we found that using a neuronal population-based approach to identify engram cells in our simulations yielded analogous engram dynamics (compare Extended Data Fig. 4j–o to Fig. 1e−j and Extended Data Fig. 2i–n to Extended Data Fig. 2b−g; Methods).

## Dynamic and selective fear memory engrams

**Fig. 3:**
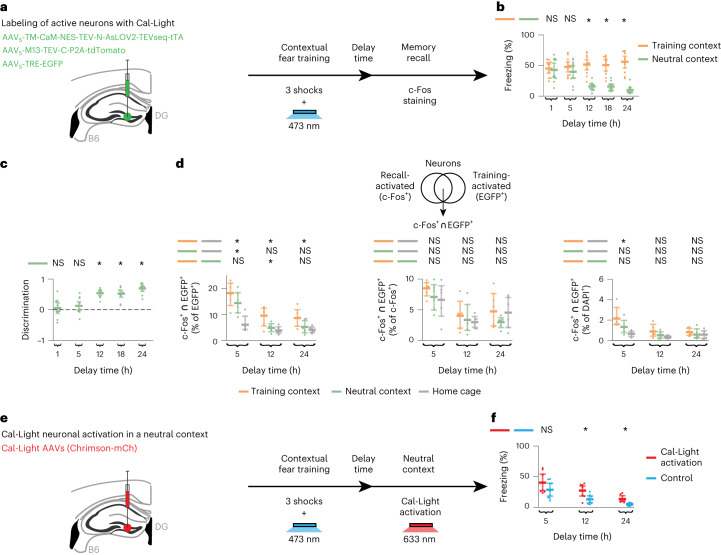
Dynamic and selective engrams encode contextual fear memory. **a**, Schematic of experimental protocol with Cal-Light labeling of fear training-activated neurons (Methods). **b**–**d**, Means and 99% confidence intervals are shown. **b**, Freezing levels during memory recall in **a** as a function of delay time. Two-sided Wilcoxon signed-rank test between freezing in the training and neutral contexts. *n* = 11 mice per group. **c**, Discrimination index between freezing levels in the training and neutral contexts in **b** as a function of delay time (Methods). Two-sided one-sample Wilcoxon signed-rank test against discrimination = 0. *n* = 11 mice per group. **d**, Ensemble overlap between neurons activated during both recall and training (*c-Fos*^+^ ∩ EGFP^+^) as a fraction of training-activated neurons (EGFP^+^) (left), recall-activated neurons (*c-Fos*^+^) (middle) and all cell counts (DAPI^+^) (right) in **a** (Methods). One-way ANOVA followed by Tukey’s HSD post hoc test. *n* = 6 mice per group. **e**, Schematic of experimental protocol with optogenetic reactivation of Cal-Light-labeled neurons (Methods). **f**, Freezing levels when mice were placed in the neutral context in **e** as a function of delay time. Means and 99% confidence intervals are shown. Unpaired *t*-test between the control and the Cal-Light activation groups. *n* = 9 mice per group. **b**–**d**,**f**, *P < 0.05; NS, not significant. For detailed statistical test results, see Supplementary Table 1. Source data

**Fig. 4:**
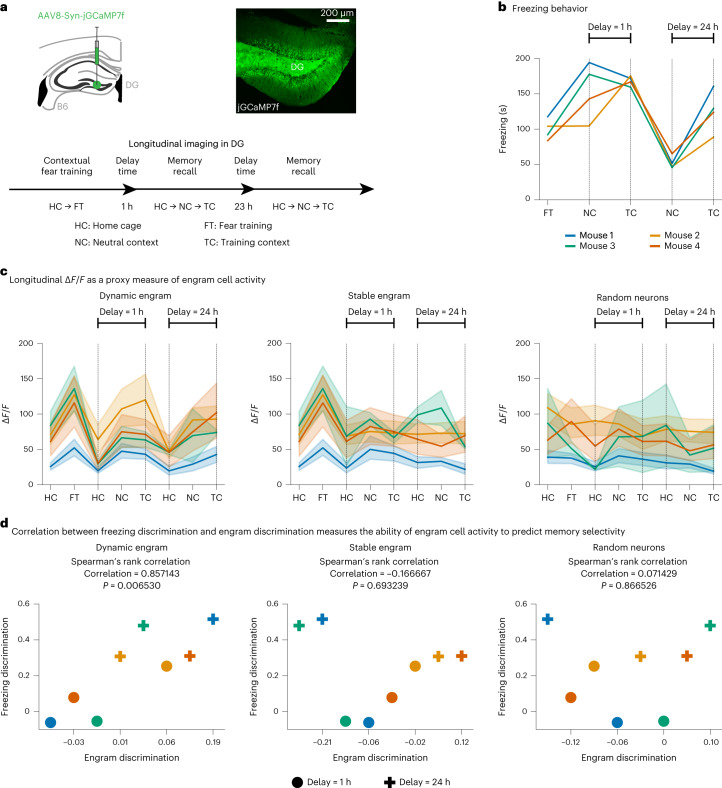
Dynamic engrams underlie the emergence of memory selectivity. **a**, Top, schematic (left) and representative image (right) of experimental mice. Bottom, schematic of experimental protocol. Representative image of a group of three independent samples. **b**, Freezing levels during fear training and memory recall. Freezing during recall in the neutral context and the training context shown for different delay times in the protocol in **a**. **c**, Average Δ*F*/*F* signal of engram cells during each session of the protocol in **a**. From left to right: dynamic engram, stable engram and random neurons. Experimental data: longitudinal Δ*F*/*F* calcium signals of imaged neurons, thus providing a proxy measure of their in vivo neural activity. Color denotes individual mouse as in **b**. Means and 95% confidence intervals are shown. **d**, Correlation between freezing discrimination and engram discrimination at different delay times. From left to right: dynamic engram, stable engram and random neurons. Discrimination indices were computed based on either the animal’s freezing levels or the average Δ*F*/*F* signals of engram cells during recall in the training and the neutral contexts (Methods). Color denotes individual mouse as in **b**. Spearman’s rank correlation coefficient and associated two-sided test with *P* are shown. Source data

**Extended Data Fig. 8:**
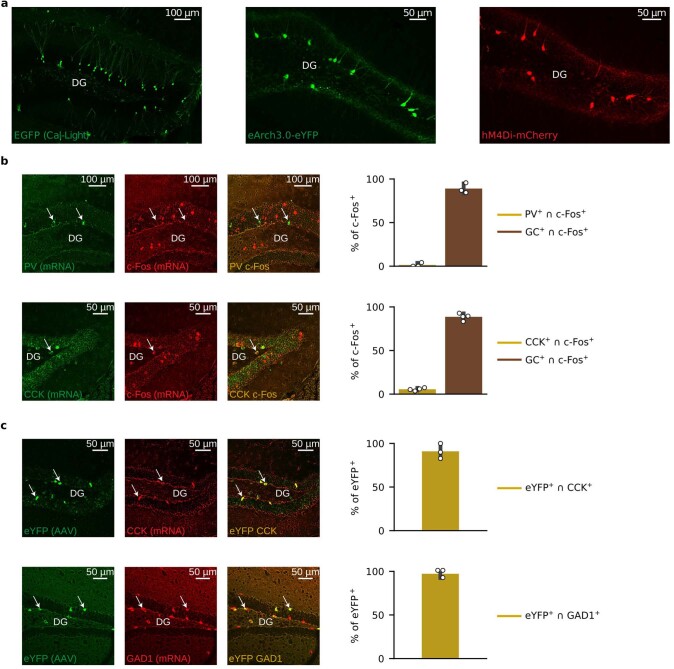
Representative images and specificity of the labeling approaches used in mouse experiments. **a**, Representative images of experimental approaches. Left, Cal-Light labeling in Fig. 3a. Middle, CCK^+^ neuronal eArch3.0-eYFP labeling in Fig. 5e. Right, CCK^+^ neuronal hM4Di-mCherry labeling in Fig. 6e. For each experiment, representative image of a group of 3 independent samples. **b-c**, Means and standard deviations are shown. **b**, Proportion of PV^+^ cells, CCK^+^ cells, and granule cells (GC^+^) in *c*-*Fos*^+^ populations. Fluorescent in situ hybridization (FISH) was used to determine the proportion of PV^+^ vs. GC^+^ cells (top row, *n* = 3 mice) and CCK^+^ vs. GC^+^ cells (bottom row, *n* = 4 mice) in *c*-*Fos*^+^ populations that were induced by contextual fear training. *c*-*Fos*^+^ labeling within the GC layer that was PV^-^ and CCK^-^ represents GC^+^ counts. Left, representative images. Right, proportion of cells in *c*-*Fos*^+^ populations. **c**, Specificity of AAV_5_-Dlx5/6-DIO-eArch3.0-eYFP in CCK-Cre mice (from Fig. 5e). FISH was used to measure the proportion of the virally-labeled CCK^+^ neurons (eYFP probe) that overlapped with endogenous CCK mRNA (top row) and endogenous GAD1 mRNA (bottom row). *n* = 3 mice per group. Source data

**Extended Data Fig. 9:**
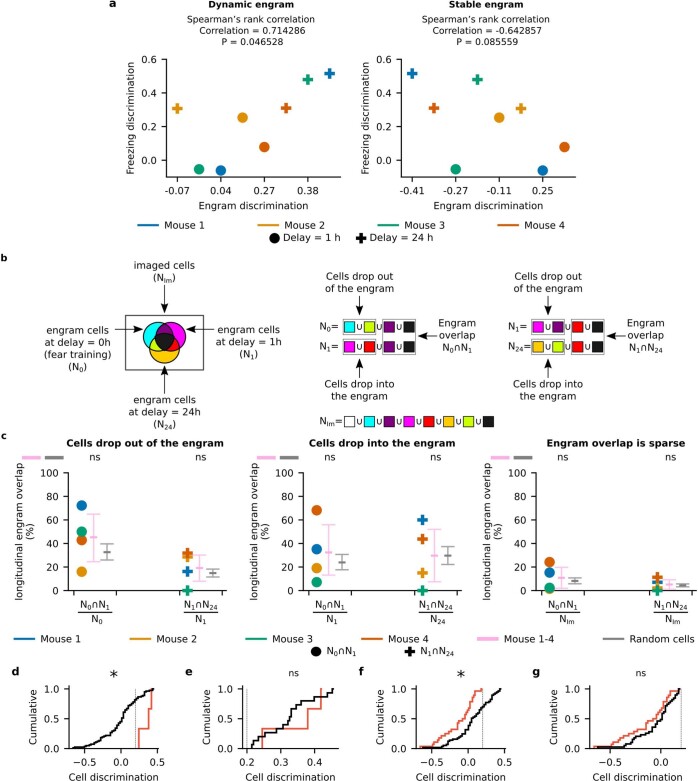
Analysis of longitudinal calcium imaging experiments. **a**, Correlation between freezing discrimination and engram discrimination at different delay times when using non-negative matrix factorization to identify engram cells in the longitudinal calcium imaging experiments in Fig. 4a (see Methods). Left, dynamic engram. Right, stable engram. Discrimination indices computed based on either the animal’s freezing levels or the average Δ*F*/*F* signals of engram cells during recall in the training and the neutral contexts (see Methods). Spearman’s rank correlation coefficient and associated two-sided test with *P* are shown. **b-c**, Longitudinal imaging reveals engram cell turnover in contextual fear memory. Analysis of engram cell ensembles identified in the longitudinal calcium imaging experiments in Fig. 4a (see Methods). **b**, Schematic depicting the set of imaged cells (N_Im_), the set of engram cells identified at delay = 0 h (that is, fear training) (N_0_), the set of engram cells identified at delay = 1 h (N_1_) and the set of engram cells identified at delay = 24 h (N_24_). By comparing N_0_ and N_1_, we can identify cells that dropped out of the engram, cells that dropped into the engram, and cells that remained in the engram (that is, engram overlap N_0_ ∩ N_1_) from delay = 0 to 1 h. By comparing N_1_ and N_24_, we can identify cells that dropped out of the engram, cells that dropped into the engram, and cells that remained in the engram (that is, engram overlap N_1_ ∩ N_24_) from delay = 1 to 24 h. **c**, Longitudinal engram overlap from delay = 0 to 1 h (that is, N_0_ ∩ N_1_) and from delay = 1 to 24 h (that is, N_1_ ∩ N_24_). Left, (N_0_ ∩ N_1_) / N_0_ measures the fraction of engram cells at delay = 0 h that remained part of the engram at delay = 1 h and (N_1_ ∩ N_24_) / N_1_ measures the fraction of engram cells at delay = 1 h that remained part of the engram at delay = 24 h. Middle, (N_0_ ∩ N_1_) / N_1_ measures the fraction of engram cells at delay = 1 h that were also part of the engram at delay = 0 h and (N_1_ ∩ N_24_) / N_24_ measures the fraction of engram cells at delay = 24 h that were also part of the engram at delay = 1 h. Right, (N_0_ ∩ N_1_) / N_Im_ measures the fraction of imaged cells that were engram cells both at delay = 0 and 1 h and (N_1_ ∩ N_24_) / N_Im_ measures the fraction of imaged cells that were engram cells both at delay = 1 and 24 h. To compare longitudinal engram overlap in mouse 1-4 to overlap at random, we generated ensembles of random cells using the following procedure. First, we took N_Im_ of an individual mouse and randomly drew ${{{{\rm{N}}}}}_{0}^{{{{\rm{random}}}}}$, ${{{{\rm{N}}}}}_{1}^{{{{\rm{random}}}}}$ and ${{{{\rm{N}}}}}_{24}^{{{{\rm{random}}}}}$ of the same size as N_0_, N_1_ and N_24_ of the same mouse, respectively. We then repeated this procedure 10 times for each mouse. Finally, we compared longitudinal engram overlap in mouse 1-4 versus random cells using a two-sided Mann-Whitney U test. (N_0_ ∩ N_1_) / N_0_, *U* = 108.5, *P* = 0.250176. (N_1_ ∩ N_24_) / N_1_, *U* = 101.5, *P* = 0.389608. (N_0_ ∩ N_1_) / N_1_, *U* = 99.5, *P* = 0.436077. (N_1_ ∩ N_24_) / N_24_, *U* = 82.5, *P* = 0.934642. (N_0_ ∩ N_1_) / N_Im_, *U* = 97.5, *P* = 0.486051. (N_1_ ∩ N_24_) / N_Im_, *U* = 86.5, *P* = 0.805871. *n* = 4 mice. For mouse 1-4, individual data points as well as mean and 95% confidence intervals are shown. For random cells, mean and 95% confidence intervals are shown. Data compared using a Mann-Whitney U test met required assumptions (that is, continuous dependent variable, independent variable consisting of two independent groups, and independence of observations). **d-g**, Analysis of the distribution of Δ*F*/*F*-based discrimination indices of imaged cells in the longitudinal calcium imaging experiments in Fig. 4a (see Methods). Representative animal shown. **d**, Cumulative distributions of Δ*F*/*F*-based discrimination indices of imaged cells in the fear training session in Fig. 4a (dashed line indicates threshold ${\zeta }_{disc}^{thr}$ = 0.2 for engram cell identification). Red: engram cells identified during fear training that remained part of the engram in all imaging sessions. Black: all other imaged cells. Two-sided Kolmogorov-Smirnov test, *P* = 0.009213. **e**, Cumulative distributions of Δ*F*/*F*-based discrimination indices of imaged cells in the fear training session in Fig. 4a (dashed line indicates threshold ${\zeta }_{disc}^{thr}$ = 0.2 for engram cell identification). Red: engram cells identified during fear training that remained part of the engram in all imaging sessions. Black: engram cells identified during fear training that dropped out of the engram in any of the imaging sessions. Two-sided Kolmogorov-Smirnov test, *P* = 0.571078. **f**, Cumulative distributions of Δ*F*/*F*-based discrimination indices of imaged cells in the fear training session in Fig. 4a (dashed line indicates threshold ${\zeta }_{disc}^{thr}$ = 0.2 for engram cell identification). Red: cells that were not identified as engram cells during fear training but dropped into the engram in any of the remaining imaging sessions. Black: all other imaged cells. Two-sided Kolmogorov-Smirnov test, *P* = 0.001345. **g**, Cumulative distributions of Δ*F*/*F*-based discrimination indices of imaged cells in the fear training session in Fig. 4a (dashed line indicates threshold ${\zeta }_{disc}^{thr}$ = 0.2 for engram cell identification). Red: cells that were not identified as engram cells during fear training but dropped into the engram in any of the remaining imaging sessions. Black: cells that were not identified as engram cells during fear training and remained non-engram cells in all imaging sessions. Two-sided Kolmogorov-Smirnov test, *P* = 0.410507. **c–g**, *P < 0.05; ns, not significant.

To test our model’s prediction that memories switch from unselective to selective as the composition of the underlying engram changes, we used a CFC behavioral paradigm. Specifically, we initially subjected mice to CFC in a training context (Fig. 3a and Methods). After a delay period following CFC, mice were initially placed in a neutral context and subsequently in the training context to evaluate memory recall by measuring freezing levels in each context. We performed recall sessions first in the neutral context and subsequently in the training context to avoid a potential confound: we needed to measure behavioral performance at short timescales after encoding (that is, delay = 1 h and 5 h), and, after a recall session in the training context, an animal’s behavioral state is thought to be altered for tens of minutes to a few hours (for example, through elevated stress hormone levels^15,16^). Freezing levels in the training and neutral contexts were similar shortly after fear training (that is, delay = 1 h and 5 h), but they differed for longer delay periods (that is, delay = 12 h, 18 h and 24 h) (Fig. 3b). By defining a discrimination index between the freezing behavior in the training and neutral contexts analogous to the one for memory recall in our simulations (Methods), we saw that memory selectivity emerged after a delay of 12 h following training (Fig. 3c). These findings were consistent with our modeling predictions (compare Fig. 3b,c to Fig. 1i,j) as well as with recent reports that conditioned taste aversion memories become selective in the timescale of hours^12^. To track the post-encoding evolution of fear memory engrams during behavior, we used the Cal-Light dual-protein switch system that translates neuronal activity-mediated calcium increases into gene expression in a light-dependent manner^17^. We injected Cal-Light, a cocktail of three adeno-associated viruses (AAVs), into the hippocampal DG of wild-type B6 mice, followed by optic fiber implants targeting DG (Fig. 3a, left illustration, and Methods). By applying blue light in vivo during fear training, we tagged training-activated DG neurons with EGFP (Fig. 3a, right illustration, and Extended Data Fig. 8a, left panel). After a delay period, we used immediate early gene *c-Fos* staining to label recall-activated neurons in the same mice. Note that activity-dependent labeling using either Cal-Light or *c-Fos* staining visualizes neurons that were highly activated during defined time windows (that is, training and recall, respectively) without providing a quantitative measure of neuronal activity. In situ hybridization confirmed that the majority of *c-Fos*^+^ cells in DG are granule cells (∼88%), whereas parvalbumin-expressing (PV^+^) and cholecystokinin-expressing (CCK^+^) cells represent only small fractions (1.39% and 5.56%, respectively) (Extended Data Fig. 8b). We observed that, when mice were returned to the training context for recall, each of the following ratios decreased over time: ensemble overlap between neurons activated during recall and training (*c-Fos*^+^ ∩ EGFP^+^) as a fraction of training-activated neurons (EGFP^+^), recall-activated neurons (*c-Fos*^+^) and all cell counts (DAPI^+^) (Fig. 3d). The decline in the level of recall-induced reactivation of training-activated neurons was consistent with our prediction that only a fraction of training-activated engram cells remain in the engram as memory consolidation progresses (Fig. 1e). The drop in the proportion of recall-activated neurons that were also active during training was in line with our prediction that new neurons are recruited into the engram after memory encoding (Fig. 1e). The decrease in the ensemble overlap between recall-activated and training-activated neurons relative to all cell counts was also consistent with our modeling results (Fig. 1e). *c-Fos*^+^ ∩ EGFP^+^ as a fraction of EGFP^+^, *c-Fos*^+^ and DAPI^+^ also dropped over time when mice were placed in the neutral context after fear training (Fig. 3d). To determine baseline levels for these ensemble overlap ratios, we measured them when mice were placed in their home cages after conditioning (Fig. 3d). Critically, *c-Fos*^+^ ∩ EGFP^+^ as a fraction of EGFP^+^ was above the home cage baseline for recall tests in the training and neutral contexts at delay = 5 h, but it was above baseline only for recall in the training context at delay = 12 h (Fig. 3d). This coincided with the emergence of fear memory selectivity (Fig. 3c), consistent with our network simulations (Fig. 1h–j). Notably, *c-Fos*^+^ ∩ EGFP^+^ as a fraction of EGFP^+^ for recall in the training context remained above the home cage baseline at delay = 24 h. We did not observe such differences between recall in the training and neutral contexts relative to the baseline in the case of *c-Fos*^+^ ∩ EGFP^+^ as a fraction of *c-Fos*^+^ or DAPI^+^ cells. This could indicate that the rate of engram cell turnover was elevated such that it would require highly precise measurements to capture differences in the fraction of recall-activated neurons that were also active during encoding and in the fraction of neurons that were active during both recall and encoding when mice are placed in the training context versus the home cage. To directly investigate the relationship between engram cell turnover and the emergence of memory selectivity, we performed a longitudinal imaging experiment as discussed below (Fig. 4 and Extended Data Fig. 9). Altogether, our experimental results supported our model’s prediction that memory consolidation leads to dynamic and selective engrams.

We next tested whether fear training-activated DG neurons labeled using Cal-Light remain sufficient for inducing recall after initial memory encoding in line with previous experimental findings^4^ and our modeling results (Fig. 2b). In particular, we activated Cal-Light-labeled DG neurons in the neutral context following a delay = 5 h, 12 h and 24 h after training (Fig. 3e). For these experiments, we replaced the TRE-EGFP virus with a TRE-Chrimson-mCherry virus in the Cal-Light three-AAV cocktail for surgeries. This allowed us to first label active neurons during training using blue light followed by red light reactivation of Cal-Light-labeled DG neurons in the neutral context. Although we found increased freezing levels (that is, memory recall) in the Chrimson-mCherry Cal-Light activation group as compared to the mCherry-alone control group across delay times, the increase was statistically significant only at delay = 12 h and 24 h (Fig. 3f). One reason why we did not observe a significant increase in freezing in the Chrimson-mCherry group at delay = 5 h was that, at this time point after fear training, control animals showed heightened levels of freezing even in the neutral context (that is, memory was still unselective at delay = 5 h; Fig. 3b). However, this was not the case for delay = 12 h and 24 h (that is, memory became selective at delay = 12 h and 24 h; Fig. 3b). Thus, these optogenetic reactivation experiments provided functional evidence for the tagging of a behavioral experience using the Cal-Light approach and demonstrated that Cal-Light-labeled DG neurons remain sufficient for inducing recall after fear training.

We also conducted longitudinal imaging experiments to directly evaluate whether the emergence of memory selectivity is related to changes in engram ensemble composition as predicted by our model. Specifically, we performed in vivo calcium imaging from neurons in the hippocampal DG of mice over the course of a CFC protocol (Fig. 4a; 461 cells and four mice). We conducted a total of eight imaging sessions at three time points: home cage and fear training; for delay = 1 h, home cage, neutral context and training context; and for delay = 24 h, home cage, neutral context and training context. We also measured freezing levels during fear training and memory recall in the neutral and training contexts for delay = 1 h and 24 h (Fig. 4b). Considering the dynamic engram hypothesis (that is, a continuously evolving engram cell ensemble), we identified engram cells at three different time points (that is, fear training, delay = 1 h and delay = 24 h) by computing a discrimination index between the average calcium signal (Δ*F*/*F*) of each imaged cell in the training context and the home cage as a normalized measure of neuronal activation (Methods). A cell was identified as an engram cell at a given time point if its Δ*F*/*F*-based discrimination index exceeded the threshold ${\zeta }_{disc}^{thr}=0.2$. We then tracked the average Δ*F*/*F* signal of the identified engram cells in each session of the CFC protocol (Fig. 4c, left panel). Next, we computed a freezing discrimination index between freezing levels during recall in the training and neutral contexts as well as an engram discrimination index between average Δ*F*/*F* signals of engram cells during recall in the training and neutral contexts (Methods). We found that freezing discrimination and engram discrimination were correlated when considering dynamic engrams (Fig. 4d, left panel). We repeated this analysis considering the stable engram hypothesis (that is, a static engram cell ensemble identified during fear training) and a random population of neurons (that is, a randomly chosen neuronal ensemble of the same size as the stable engram ensemble) but found no correlation between freezing discrimination and engram discrimination in either case (Fig. 4c,d, middle and right panels). When using a neuronal population-based approach to identify engram cells, we also found that freezing discrimination and engram discrimination were correlated considering dynamic but not stable engrams (Extended Data Fig. 9a and Methods). Hence, our results provide evidence that the activity of dynamic engrams, but not stable engrams or random neurons, is predictive of an animal’s memory discrimination ability. Furthermore, we tracked the longitudinal overlap between engram cell ensembles identified during consecutive time points in our imaging experiments to characterize how the composition of the engram evolved after memory encoding (Extended Data Fig. 9b,c). We found that neurons dropped out of (Extended Data Fig. 9c, left panel) and dropped into (Extended Data Fig. 9c, middle panel) the engram over the course of memory consolidation with sparse levels of engram ensemble overlap (Extended Data Fig. 9c, right panel). Notably, we did not observe a significant difference between longitudinal engram overlap in our mouse imaging data and overlap at random (Extended Data Fig. 9c), but these results are preliminary given the small sample size (*n* = 4 mice). To evaluate whether Δ*F*/*F* calcium signals during memory encoding were predictive of neurons dropping out of or dropping into the engram, we examined the distribution of Δ*F*/*F*-based discrimination indices during fear training (Extended Data Fig. 9d–g). We found that training-activated engram cells that remained part of the engram during subsequent recall sessions exhibited higher Δ*F*/*F*-based discrimination indices during fear training than the remaining imaged cells (Extended Data Fig. 9d). Although we did not observe a significant difference between Δ*F*/*F*-based discrimination indices during fear training in the case of training-activated engram cells that remained part of the engram and training-activated engram cells that dropped out of the engram after training (Extended Data Fig. 9e), this may reflect that large sample sizes would be required to capture such a difference given engram sparsity (Extended Data Fig. 9c, right panel). Thus, our findings point to Δ*F*/*F*-based discrimination indices during fear training being indicative of a neuron’s ability to remain part of the engram after initial memory encoding in a manner consistent with our modeling results (compare Extended Data Fig. 9d,e to Extended Data Fig. 3j,k). We also found that cells that were not activated during fear training but later dropped into the engram showed lower Δ*F*/*F*-based discrimination indices during fear training than the remaining imaged cells, but we did not observe such a difference relative to cells that failed to become part of the engram entirely (Extended Data Fig. 9f,g; compare to Extended Data Fig. 3l,m). Altogether, our experimental results directly captured the link between the emergence of memory selectivity and changes in engram ensemble composition in line with our model’s prediction.

## Inhibition is crucial for memory selectivity

**Fig. 5:**
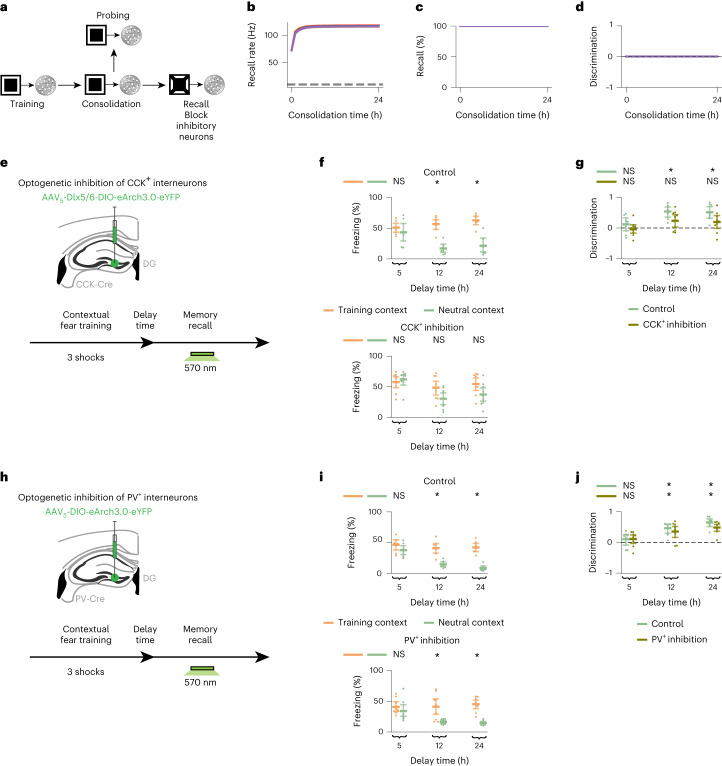
Inhibitory activity during recall is critical for memory selectivity. **a**, Schematic of simulation protocol with blockage of inhibitory neurons during recall. **b**–**d**, Means and 99% confidence intervals are shown. *n* = 10 trials. Color denotes stimulus as in Fig. 1c. **b**, Firing rate of engram cells averaged across all cue presentations during recall in **a** as a function of consolidation time (Methods). Dashed line indicates threshold *ζ*^*thr*^ = 10 Hz for engram cell activation. **c**, Memory recall in **a** as a function of consolidation time. **d**, Discrimination index between recall evoked by cues of the training stimulus and individual novel stimuli in **a** as a function of consolidation time (Methods). **e**, Schematic of experimental protocol with optogenetic inhibition of DG CCK^+^ interneurons. **f**,**g**, Means and 99% confidence intervals are shown. **f**, Freezing levels during memory recall in **e** as a function of delay time. Top, control group. Bottom, CCK^+^ inhibition group. Two-sided Wilcoxon signed-rank test between freezing in the training and neutral contexts. Control, *n* = 7 mice per group. CCK^+^ inhibition, *n* = 9 mice per group. **g**, Discrimination index between freezing levels in the training and neutral contexts in **f** (Methods). Two-sided one-sample Wilcoxon signed-rank test against discrimination = 0. Control, *n* = 7 mice per group. CCK^+^ inhibition, *n* = 9 mice per group. **h**, Schematic of experimental protocol with optogenetic inhibition of DG PV^+^ interneurons. **i**,**j**, Means and 99% confidence intervals are shown. **i**, Freezing levels during memory recall in **h** as a function of delay time. Top, control group. Bottom, PV^+^ inhibition group. Two-sided Wilcoxon signed-rank test between freezing in the training and neutral contexts. Control, *n* = 7 mice per group. PV^+^ inhibition, *n* = 9 mice per group. **j**, Discrimination index between freezing levels in the training and neutral contexts in **i**. Two-sided one-sample Wilcoxon signed-rank test against discrimination = 0. Control, *n* = 7 mice per group. PV^+^ inhibition, *n* = 9 mice per group. **f**,**g**,**i**,**j**, *P < 0.05; NS, not significant. For detailed statistical test results, see Supplementary Table 1. Source data

**Extended Data Fig. 10:**
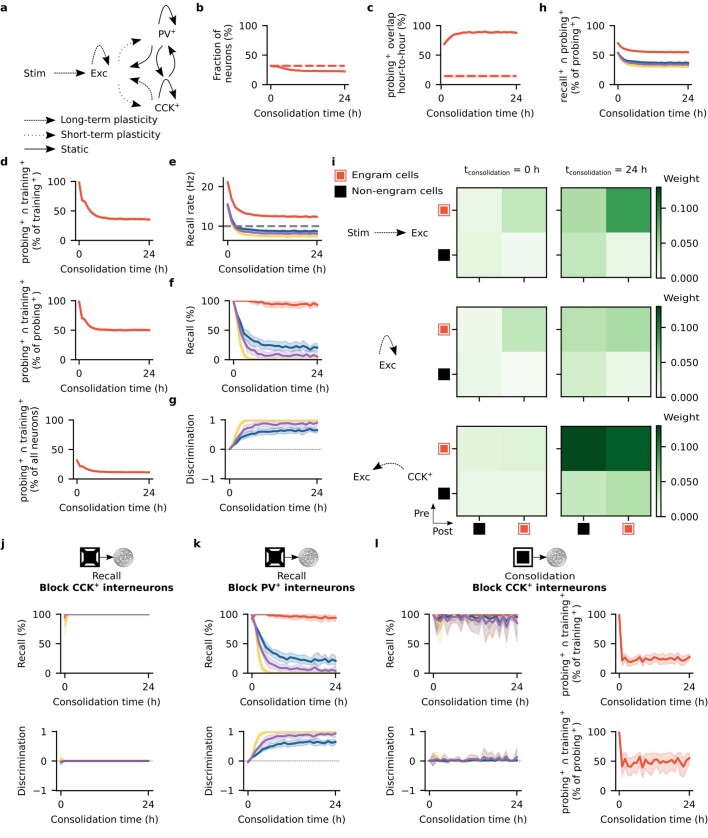
CCK^+^ interneurons support memory selectivity. **a**, Schematic of network model with CCK^+^ and PV^+^ interneurons. The network consists of a stimulus population (Stim) and a hippocampus network similar to Fig. 1a but with excitatory neurons (Exc) as well as CCK^+^ and PV^+^ interneurons. Plasticity of feedforward and recurrent synapses are shown. Specifically, feedforward excitatory synapses and recurrent excitatory synapses onto excitatory neurons exhibit short- and long-term excitatory synaptic plasticity, whereas recurrent excitatory synapses onto CCK^+^ or PV^+^ interneurons only display short-term plasticity. CCK^+^ synapses onto excitatory neurons are subject to inhibitory synaptic plasticity (Equation 15) while the remaining inhibitory synapses are static (see Methods). **b-i**, Simulation protocol as in Fig. 1b with the network in **a**. **b-h**, Means and 99% confidence intervals are shown. *n* = 10 trials. **b**, Engram cell ensemble as a fraction of all neurons. Dashed line indicates engram cell ensemble at the end of training. **c**, Ensemble overlap between probing-activated engram cells at consolidation time = *t* and *t*-1 h as a fraction of engram cells at consolidation time = *t*-1 h. Dashed line indicates ensemble of neurons that remained part of the engram in all sampled time points (that is, consolidation time = 0, 1, …, 24 h) as a fraction of engram cells at consolidation time = 0 h (that is, training-activated engram cells). **d**, Ensemble overlap between engram cells activated during both probing and training as a fraction of training-activated engram cells (top), probing-activated engram cells (middle) and all neurons in the network (bottom). **e**, Firing rate of engram cells averaged across all cue presentations during recall as a function of consolidation time. Dashed line indicates threshold *ζ*^*thr*^ = 10 Hz for engram cell activation. **f**, Memory recall as a function of consolidation time. **g**, Discrimination index between recall evoked by cues of the training stimulus and individual novel stimuli as a function of consolidation time (see Methods). **h**, Fraction of probing-activated engram cells reactivated during recall as a function of consolidation time. **i**, Mean weight strength of plastic synapses clustered according to engram cell status. Top, feedforward excitatory synapses onto excitatory neurons. Middle, recurrent excitatory synapses onto excitatory neurons. Bottom, recurrent CCK^+^ synapses onto excitatory neurons. Left, at the end of the training phase. Right, after 24 h of consolidation. Representative trial shown. **j-l**, Means and 99% confidence intervals are shown. *n* = 10 trials. **j**, Simulation protocol as in Fig. 1b with the network in **a** but with CCK^+^ interneurons blocked during recall. Top, memory recall as a function of consolidation time. Bottom, discrimination index between recall evoked by cues of the training stimulus and individual novel stimuli as a function of consolidation time (see Methods). **k**, Simulation protocol as in Fig. 1b with the network in **a** but with PV^+^ interneurons blocked during recall. Top, memory recall as a function of consolidation time. Bottom, discrimination index between recall evoked by cues of the training stimulus and individual novel stimuli as a function of consolidation time (see Methods). **l**, Simulation protocol as in Fig. 1b with the network in **a** but with CCK^+^ interneurons blocked during consolidation. This also blocked the plasticity of CCK^+^ synapses onto excitatory neurons during consolidation (see Methods). The remaining CCK^+^ synapses continued to be static in the entire simulation protocol as in **a**. Left, memory recall as a function of consolidation time (top) and discrimination index between recall evoked by cues of the training stimulus and individual novel stimuli as a function of consolidation time (bottom) (see Methods). Right, ensemble overlap between engram cells activated during both probing and training as a fraction of training-activated engram cells (top) and probing-activated engram cells (bottom). **e-h/j-l**, Color denotes stimulus as in Fig. 1c. **a-l**, Network simulation parameters as in Fig. 1a-c except that $${N}_{CC{K}^{+}}={N}_{P{V}^{+}}=512$$ interneurons, $${w}^{E\to CC{K}^{+}}={w}^{E\to P{V}^{+}}=0.6,{w}^{CC{K}^{+}\to CC{K}^{+}}$$ $$={w}^{P{V}^{+}\to P{V}^{+}}=0.2,{w}^{CC{K}^{+}\to E}={w}^{P{V}^{+}\to E}=0.2,{w}^{CC{K}^{+}\to P{V}^{+}}={w}^{P{V}^{+}\to CC{K}^{+}}$$$$=0.02,{\alpha }^{CC{K}^{+}}={\alpha }^{P{V}^{+}}=0.3,{\nu }_{training}^{stim}$$ $$={\nu }_{probing}^{stim}=15\,{{{\rm{Hz}}}},{\nu }_{consolidation}^{stim}=30\,{{{\rm{Hz}}}}$$ and $${\nu }_{recall}^{stim}=20\,{{{\rm{Hz}}}}$$ (see Methods and Supplementary Table 2).

We next examined the role of inhibition in memory selectivity using our network model. We blocked inhibitory neurons during memory recall, and this disrupted selectivity in our simulations (Fig. 5a–d; see Extended Data Fig. 7i for a simulation with an alternative form of inhibitory synaptic plasticity). Previous experiments showed that inhibiting DG CCK^+^ interneurons during recall 24 h or 48 h after CFC impairs memory selectivity, but inhibiting DG PV^+^ interneurons during recall at the same time points has no effect on selectivity^5^. This was associated with an enhanced post-training inhibitory synaptic input from DG CCK^+^ but not PV^+^ interneurons onto training-activated engram cells^5^. To capture this difference between interneuron types in mediating memory selectivity, we expanded our network model to include both CCK^+^ and PV^+^ interneurons (Extended Data Fig. 10a). CCK^+^ but not PV^+^ synapses onto excitatory neurons were plastic, consistent with experimental reports^5^. Dynamic and selective engrams emerged in the expanded model in a manner analogous to our original network (compare Extended Data Fig. 10b–i to Fig. 1e–l). In addition, blocking CCK^+^ interneurons during recall disrupted memory selectivity, but blocking PV^+^ interneurons during recall had no such impact (Extended Data Fig. 10j,k), in line with experimental results^5^. Note that blocking either CCK^+^ or PV^+^ interneurons before the emergence of selectivity (that is, at consolidation time = 0 h) had a negligible effect on memory recall and discrimination (compare Extended Data Fig. 10j,k to Extended Data Fig. 10f,g). Thus, our model predicted that inhibitory neurons—and specifically DG CCK^+^ but not PV^+^ interneurons—are essential for expressing memory selectivity once engrams have become selective. To experimentally test this prediction, we first used CCK-Cre mice injected with a Dlx5/6-driven Cre-dependent eArch3.0-eYFP virus in DG to optogenetically inhibit (by applying green light in vivo) DG CCK^+^ interneurons during memory recall 5 h, 12 h or 24 h after contextual fear training (Fig. 5e and Extended Data Fig. 8a, middle panel; Methods). Using in situ hybridization, we confirmed that this labeling approach results in 90.95% of the eYFP-labeled DG cells corresponding to CCK^+^ cells, which are also inhibitory in nature as 97.22% of the eYFP-labeled DG cells expressed GAD1 (Extended Data Fig. 8c). Mice in the CCK^+^ inhibition group were unable to discriminate between the training and neutral contexts at all tested delay times, whereas those in the control group displayed memory selectivity with delay times of 12 h and 24 h (Fig. 5f,g; compare to Fig 3b,c). Next, we performed similar optogenetic manipulation experiments using PV-Cre mice injected with a Cre-dependent eArch3.0-eYFP virus in DG (Fig. 5h and Methods). We found that mice in both the PV^+^ inhibition and control groups developed the ability to discriminate between the training and neutral contexts at delay times of 12 h and 24 h (Fig. 5i,j). Therefore, our experimental findings demonstrated that DG CCK^+^ but not PV^+^ interneurons are required for memory discrimination, and, hence, they supported our model’s prediction that cell-type-specific inhibitory activity during recall is critical for engram selectivity.

## Inhibitory synaptic plasticity molds selective engrams

**Fig. 6:**
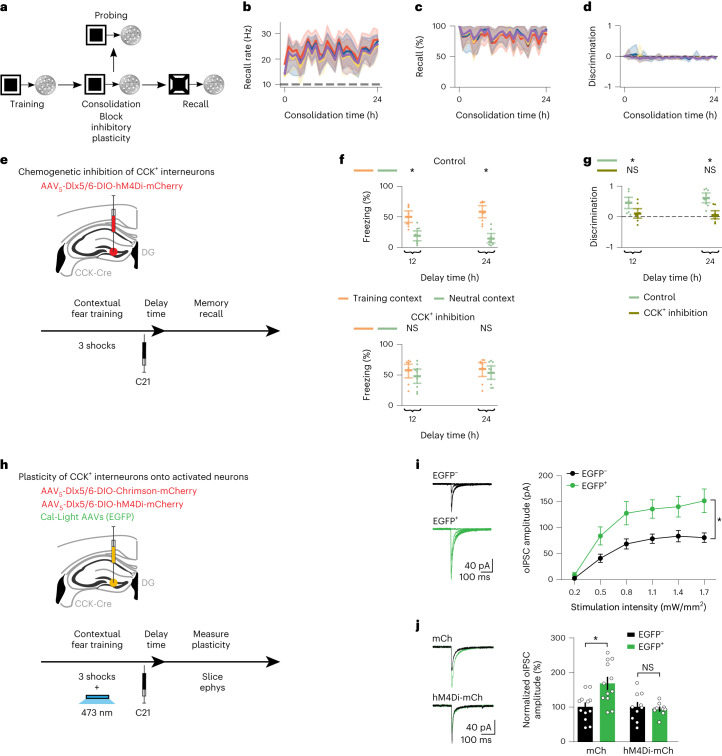
Inhibitory synaptic plasticity during memory consolidation carves selective engrams. **a**, Schematic of simulation protocol with blockage of inhibitory synaptic plasticity during consolidation. **b**–**d**, Means and 99% confidence intervals are shown. *n* = 10 trials. Color denotes stimulus as in Fig. 1c. **b**, Firing rate of engram cells averaged across all cue presentations during recall in **a** as a function of consolidation time (Methods). Dashed line indicates threshold *ζ*^*thr*^ = 10 Hz for engram cell activation. **c**, Memory recall in **a** as a function of consolidation time. **d**, Discrimination index between recall evoked by cues of the training stimulus and individual novel stimuli in **a** as a function of consolidation time (Methods). **e**, Schematic of experimental protocol with chemogenetic inhibition of DG CCK^+^ interneurons. **f**,**g**, Means and 99% confidence intervals are shown. **f**, Freezing levels during memory recall in **e** as a function of delay time. Top, control group. Bottom, CCK^+^ inhibition group. Two-sided Wilcoxon signed-rank test between freezing in the training and neutral contexts. Control, *n* = 9 mice per group. CCK^+^ inhibition, *n* = 9 mice per group. **g**, Discrimination index between freezing levels in the training and neutral contexts in **f**. Two-sided one-sample Wilcoxon signed-rank test against discrimination = 0. Control, *n* = 9 mice per group. CCK^+^ inhibition, *n* = 9 mice per group. **h**, Schematic of experimental protocol to measure the plasticity of DG CCK^+^ efferent synapses onto Cal-Light-labeled EGFP^+^ neurons using slice recordings. Ephys, electrophysiology. **i**,**j**, Means and s.e.m. are shown. **i**, oIPSCs recorded in **h**. Left, representative traces showing oIPSCs recorded in neighboring EGFP^+^ and EGFP^−^ neurons. Right, comparison of oIPSC amplitudes between EGFP^+^ and EGFP^−^ neurons. Two-way repeated-measures ANOVA with Greenhouse–Geisser correction. For EGFP^+^ versus EGFP^−^, *n* = 12 neurons per group from three mice. **j**, oIPSCs recorded in the control (mCh) and CCK^+^ inhibition (hM4Di-mCh) groups in **h**. Left, representative traces showing oIPSCs recorded in neighboring EGFP^+^ and EGFP^−^ neurons. Right, comparison of normalized oIPSC amplitudes between EGFP^+^ and EGFP^−^ neurons. Paired *t*-test. mCh group, *n* = 12 neurons per group from three mice. hM4Di-mCh group, *n* = 10 neurons per group from three mice. **f**,**g**,**i**,**j**, *P < 0.05; NS, not significant. For detailed statistical test results, see Supplementary Table 1. Source data

Given the essential role of inhibition in the expression of memory selectivity, we investigated the contribution of post-encoding inhibitory synaptic plasticity to the emergence of selective engrams. To that end, we blocked inhibitory synaptic plasticity exclusively during memory consolidation in our network simulations, and this impaired memory selectivity (Fig. 6a–d, and see Extended Data Fig. 7j for a simulation with an alternative form of inhibitory synaptic plasticity). Note that we previously blocked inhibitory neurons during recall (Fig. 5a), but here we blocked inhibitory synaptic plasticity only during memory consolidation (Fig. 6a). Furthermore, we specifically blocked CCK^+^ interneurons during memory consolidation in our expanded network model containing both CCK^+^ and PV^+^ interneurons, and this disrupted memory selectivity (Extended Data Fig. 10l). Critically, blocking CCK^+^ interneurons in our simulations also blocked the plasticity of their efferent synapses given that inhibitory synaptic plasticity in our model required pre-synaptic activity (Methods), in line with several reports that coincident pre-synaptic and post-synaptic activity as well as pre-synaptic activity alone can induce plasticity of *γ*-aminobutyric acid-releasing (GABAergic) synapses onto excitatory neurons^18^. Interestingly, engram cell turnover was still present in these simulations despite the absence of inhibitory plasticity (Extended Data Fig. 10l, right panels). This suggested that excitatory synaptic plasticity alone can drive the emergence of dynamic engrams—consistent with our previous results showing that blocking LTP during memory consolidation leads to engram stabilization (Extended Data Fig. 4a–d). Notably, our previous modeling results showing that CCK^+^ but not PV^+^ interneurons are necessary for expressing memory selectivity during recall (Extended Data Fig. 10j,k) already suggested that inhibitory synaptic plasticity has a critical role in the development of memory selectivity given that CCK^+^ but not PV^+^ efferent synapses onto excitatory neurons exhibited plasticity (Extended Data Fig. 10a). Therefore, our network model predicted that blocking inhibitory synaptic plasticity during memory consolidation prevents the emergence of engram selectivity.

To experimentally test this prediction, we used a chemogenetic approach to temporarily inhibit DG CCK^+^ interneurons right after fear training (Fig. 6e and Extended Data Fig. 8a, right panel; Methods). Specifically, we injected a Dlx5/6-driven Cre-dependent hM4Di-mCherry virus in the DG of CCK-Cre mice followed by an intraperitoneal injection of C21 immediately after fear training, hence inhibiting CCK^+^ interneurons for the first several hours of memory consolidation. Once the effects of the chemogenetic manipulation had subsided (that is, at least 12 h later), we conducted recall tests and observed that mice in the CCK^+^ inhibition group failed to discriminate between the training and neutral contexts, whereas those in the control group exhibited memory selectivity (Fig. 6f,g, compare to Fig. 3b,c). Note that, previously, we optogenetically inhibited CCK^+^ interneurons only during memory recall (Fig. 5e), whereas, here, we chemogenetically inhibited CCK^+^ interneurons immediately after fear training (that is, during the cellular consolidation phase) (Fig. 6e). To determine whether the CCK^+^ chemogenetic inhibition protocol blocks CCK^+^ plasticity onto DG training-activated engram cells, we performed a slice recording experiment in CCK-Cre mice that were injected with Cal-Light AAVs expressing EGFP, a Dlx5/6-driven Cre-dependent hM4Di-mCherry virus and a Dlx5/6-driven Cre-dependent Chrimson-mCherry virus (Fig. 6h and Methods). Using blue light during fear training, we labeled active neurons in DG with Cal-Light. This was followed by C21 administration immediately after fear training to inhibit CCK^+^ interneurons during consolidation. After 12 h, we prepared brain slices to record optogenetically-evoked inhibitory post-synaptic currents (oIPSCs) of CCK^+^ interneurons onto EGFP^+^ versus EGFP^−^ DG cells. Although in the mCherry-alone control group we observed larger oIPSCs in EGFP^+^ cells as compared to EGFP^−^ cells, this difference was not found in the hM4Di-mCherry CCK^+^ inhibition group (Fig. 6i,j). These results showed that chemogenetically inhibiting CCK^+^ interneurons using C21 blocks the plasticity of their efferent synapses. We also examined the effect of CCK^+^ interneuron inhibition during consolidation on engram cell turnover. For this purpose, we injected CCK-Cre mice with Cal-Light AAVs expressing EGFP and a Dlx5/6-driven Cre-dependent hM4Di-mCherry virus targeting DG (Extended Data Fig. 6i). We labeled training-activated neurons using blue light, and, immediately after fear training, we administered C21 to inhibit CCK^+^ interneurons during consolidation. After a post-training delay period, we placed mice in the training context and visualized recall-activated neurons using *c-Fos* staining. Given that CCK^+^ interneuron inhibition during consolidation did not prevent the emergence of dynamic engrams (Extended Data Fig. 6j), these experiments suggested that post-encoding CCK-mediated inhibitory plasticity is not required for engram cell turnover in line with our modeling results (Extended Data Fig. 10l). Thus, our combined experimental findings supported our model’s prediction that inhibitory synaptic plasticity during memory consolidation is necessary for the emergence of selective engrams.

## Discussion

We have shown that memories are encoded by dynamic engrams that become selective with memory consolidation. Previous experiments examined the long-term temporal evolution of neuronal ensembles encoding fear memories in mouse prefrontal cortex (PFC) and reported that neurons activated during later recall sessions (14 d after training) are more robustly reactivated during remote recall (28 d after training) than neurons activated during fear training or earlier recall sessions (1 d after training)^19^. In addition, systems consolidation of fear memories in mice was found to involve training-activated PFC engram cells transitioning from an initial silent (that is, cannot be reactivated by partial cues) to an active (that is, can be reactivated by partial cues) state over roughly 12 d and training-activated DG engram cells switching from active to silent in the same timescale^20^. Furthermore, increased feedforward inhibition in the DG-CA3 circuit has been shown to promote the development of context-specific PFC neuronal ensembles at both recent and remote time points after CFC^21^. Moreover, a recent study reported that neurons in the lateral amygdala activated during an initial fear training session become dispensable for memory retrieval after retraining, although their artificial reactivation still elicits recall^22^. Our results extended these findings in several ways. First, we showed that DG engrams change in a much shorter timescale, corresponding to hours, with neurons being both removed from and added to the engram cell ensemble. Consequently, this rapid turnover of DG engram cells happens before their active-to-silent transition linked to systems consolidation and without retraining. Second, we proposed a computational framework that identified ongoing synaptic plasticity during memory consolidation as a fundamental mechanism driving changes in engram composition. Third, we found that engram cell turnover is associated with the emergence of selective engrams. Fourth, we showed that inhibition and inhibitory synaptic plasticity are required for expressing and developing memory selectivity, respectively. Lastly, our computational model was able to reconcile training-activated engram cells being necessary and sufficient for memory recall^3,4^ with a continuously changing engram.

Neural representations in the olfactory, visual, parietal, prefrontal and motor cortices as well as in the hippocampus have been shown to change or drift over time when animals are repeatedly exposed to the same perceptual, navigational, stress-inducing or sensorimotor task^23–28^. This representational drift was observed in timescales ranging from minutes to weeks depending on the brain region and experimental paradigm. Notably, recent work has shed light on how time and experience distinctly contribute to representational drift^29,30^. Although our work specifically explored changes in memory engrams within hours of initial encoding, our proposition that ongoing post-encoding synaptic plasticity drives continuous changes in neural representations may be a general unifying mechanism that could account for representational drift across regions and tasks. We also associated engram cell turnover to the emergence of memory selectivity. Therefore, our results suggest that representational drift may not necessarily be just a byproduct of synaptic plasticity but may effectively have computational and behavioral functions—in line with a proposed role of neural ensemble fluidity in memory updating and flexibility^31^. For further discussion of our findings, see the Supplementary Discussion.

Past memory models conforming to standard theories of stable memory traces have shed light on several features of network dynamics supporting memory formation and recall^9,32,33^. Our work built on these results to uncover the dynamic nature of memory engrams and the interplay between engram state and memory expression mediated by ongoing synaptic plasticity, possibly offering new directions to investigate pathological conditions characterized by persistently unselective aversive memories, such as post-traumatic stress and panic disorders.

## Methods

Our experiments complied with all relevant ethical regulations following US National Institutes of Health (NIH) guidelines and were approved by the Massachusetts Institute of Technology Department of Comparative Medicine and Committee on Animal Care.

### Neuron model

We used leaky integrate-and-fire neurons with spike frequency adaption. The membrane voltage *U*_*i*_ of a neuron *i* followed^9^:

1 $${\tau }^{m}\frac{d{U}_{i}}{dt}=({U}^{rest}-{U}_{i})+{g}_{i}^{\,exc}(t)({U}^{exc}-{U}_{i})+\left({g}_{i}^{gaba}(t)+{g}_{i}^{a}(t)\right)({U}^{inh}-{U}_{i})$$

where *τ*^*m*^ indicates the membrane time constant; *U*^*rest*^ indicates the membrane resting potential; *U*^*exc*^ indicates the excitatory reversal potential; and *U*^*inh*^ indicates the inhibitory reversal potential. The synaptic conductance terms ${g}_{i}^{\,exc}(t)$, ${g}_{i}^{\,gaba}(t)$ and ${g}_{i}^{\,a}(t)$ are discussed in the next subsection.

When the membrane voltage of a neuron *i* exceeds a threshold *ϑ*_*i*_, it fires a spike. Immediately after firing a spike, the membrane voltage of the neuron is set to ${U}_{i}^{rest}$, and its firing threshold is temporarily raised to *ϑ*^*spike*^. In the absence of further spikes, the firing threshold decays to its resting value *ϑ*^*rest*^ with time constant *τ*^*thr*^ according to:

2 $${\tau }^{thr}\frac{d{\vartheta }_{i}}{dt}={\vartheta }^{rest}-{\vartheta }_{i}$$

### Synapse model

We employed a conductance-based synaptic input model. The inhibitory synaptic input ${g}_{i}^{\,gaba}$ and spike-triggered adaption ${g}_{i}^{\,a}$ evolved following^9^:

3 $$\frac{d{g}_{i}^{\,gaba}}{dt}=-\frac{{g}_{i}^{\,gaba}}{{\tau }^{gaba}}+\mathop{\sum}\limits_{j\in inh}{w}_{ij}{S}_{j}(t)$$

4 $$\frac{d{g}_{i}^{\,a}}{dt}=-\frac{{g}_{i}^{\,a}}{{\tau }^{a}}+{\Delta }^{a}{S}_{i}(t)$$

where ${S}_{j}(t)={\sum }_{k}\delta (t-{t}_{j}^{k})$ denotes the pre-synaptic spike train, and ${S}_{i}(t)={\sum }_{k}\delta (t-{t}_{i}^{k})$ denotes the post-synaptic spike train. In both instances, *δ* indicates the Dirac delta function, and ${t}_{x}^{k}(k=1,2,\ldots )$ indicates the firing times of neuron *x*. *w*_*ij*_ indicates the weight from neuron *j* to neuron *i*. Δ^*a*^ indicates a fixed adaptation strength. *τ*^*gaba*^ indicates the GABA decay time constant, and *τ*^*a*^ indicates the adaptation time constant.

Excitatory synaptic input is set by a combination of a fast AMPA-like conductance ${g}_{i}^{\,ampa}(t)$ and a slow NMDA-like conductance ${g}_{i}^{\,nmda}(t)$:

5 $${g}_{i}^{\,exc}(t)=\alpha {g}_{i}^{\,ampa}(t)+(1-\alpha ){g}_{i}^{\,nmda}(t)$$

6 $$\frac{dg_{i}^{\,ampa}}{dt} = -\frac{g_{i}^{\,ampa}}{\tau^{ampa}} + \sum\limits_{j \in exc} w_{ij} \underbrace{u_{j}(t)x_{j}(t)}_{{\rm{Short}}-{\rm{Term}}\,{\rm{Plasticity}}} S_{j}(t)$$

7 $${\tau }^{nmda}\frac{d{g}_{i}^{\,nmda}}{dt}=-{g}_{i}^{\,nmda}+{g}_{i}^{\,ampa}$$

where *α* indicates a constant that determines the relative contribution of ${g}_{i}^{\,ampa}(t)$ and ${g}_{i}^{nmda}(t)$, and *τ*^*ampa*^ and *τ*^*nmda*^ indicate their respective time constants. *u*_*j*_(*t*) and *x*_*j*_(*t*) denote variables that represent the state of short-term plasticity as discussed in the next subsection.

### Synaptic plasticity model

We designed our synaptic plasticity model after previous work that demonstrated that a combination of Hebbian (that is, triplet STDP) and non-Hebbian (that is, heterosynaptic and transmitter-induced) forms of excitatory plasticity is able to support stable memory formation and recall in spiking neural networks^9^.

#### Short-term plasticity

The variables *u*_*j*_(*t*) and *x*_*j*_(*t*) representing the state of short-term plasticity followed^9^:

8 $$\frac{d}{dt}{x}_{j}(t)=\frac{1-{x}_{j}(t)}{{\tau }^{d}}-{u}_{j}(t){x}_{j}(t){S}_{j}(t)$$

9 $$\frac{d}{dt}{u}_{j}(t)=\frac{U-{u}_{j}(t)}{{\tau }^{f}}+U\left(1-{u}_{j}(t)\right){S}_{j}(t)$$

where *τ*^*d*^ and *τ*^*f*^ indicate the depression and facilitation time constants, respectively. The parameter *U* indicates the initial release probability.

#### Long-term excitatory synaptic plasticity

Long-term excitatory synaptic plasticity combined triplet STDP^6^, heterosynaptic plasticity^7^ and transmitter-induced plasticity^8^ by having an excitatory synaptic weight *w*_*ij*_ from an excitatory neuron *j* to another excitatory neuron *i* evolve according to^9^:

10a $$\frac{d}{dt}{w}_{ij}(t)={\eta }^{exc}\left(A{z}_{j}^{+}(t){z}_{i}^{\,slow}(t-\epsilon ){S}_{i}(t)-{B}_{i}(t){z}_{i}^{-}(t){S}_{j}(t)\right)\quad {{{\rm{triplet}}}}$$

10b $$-\beta \left({w}_{ij}-{\tilde{w}}_{ij}(t)\right){\left({z}_{i}^{-}(t-\epsilon )\right)}^{3}{S}_{i}(t)\quad {{{\rm{heterosynaptic}}}}$$

10c $$+\delta {S}_{j}(t)\quad \,{{\mbox{transmitter-induced}}}\,$$

where *η*^*exc*^ (excitatory learning rate), *A* (LTP rate), *β* (heterosynaptic plasticity strength) and *δ* (transmitter-induced plasticity strength) indicate fixed parameters. *ϵ* indicates an infinitesimal offset whose purpose is to ensure that the current action potential is disregarded in the trace. State variables ${z}_{j/i}^{x}$ indicate either pre-synaptic or post-synaptic traces, and each has independent temporal dynamics with time constant *τ*^*x*^:

11 $$\frac{d{z}_{j/i}^{\,x}}{dt}=-\frac{{z}_{j/i}^{\,x}}{{\tau }^{x}}+{S}_{j/i}(t)$$

The reference weights ${\tilde{w}}_{ij}(t)$ also display independent synaptic consolidation dynamics following the negative gradient of a double-well potential^9^:

12 $${\tau }^{\,cons}\frac{d}{dt}{\tilde{w}}_{ij}(t)={w}_{ij}(t)-{\tilde{w}}_{ij}(t)-P{\tilde{w}}_{ij}(t)\left(\frac{{w}^{P}}{2}-{\tilde{w}}_{ij}(t)\right)\left({w}^{P}-{\tilde{w}}_{ij}(t)\right)$$

where *P* and *w*^*P*^ indicate fixed parameters, and *τ*^*cons*^ indicates the synaptic consolidation time constant. *P* sets the magnitude of the double-well potential. When *w*^*P*^ = 0.5, an upper stable fixed point is located at ${w}_{ij}(t)={\tilde{w}}_{ij}(t)$, and a lower stable fixed point is located at ${\tilde{w}}_{ij}(t)=0$. If ${w}_{ij}(t) > {\tilde{w}}_{ij}(t)$ by a small margin, then the upper and lower stable fixed points of ${\tilde{w}}_{ij}(t)$ are increased. If ${w}_{ij}(t)\gg {\tilde{w}}_{ij}(t)$, then ${\tilde{w}}_{ij}(t)$ only maintains a single fixed point that has a high value. This synaptic consolidation model is consistent with previous theoretical work^34^ and with synaptic tagging experiments that found that long-lasting LTP relies on events taking place during, as well as before, its initial induction^35^. In addition, this model assumes the existence of molecular mechanisms that enable synapses to maintain a stable efficacy (that is, weight) even in the presence of intermittent fluctuations triggered by various factors (for example, molecular turnover)^36,37^. Furthermore, the LTD rate *B*_*i*_(*t*) is subject to homeostatic regulation following:

13 $${B}_{i}(t)=\left\{\begin{array}{ll}A{C}_{i}(t)\quad &{{{\rm{for}}}}\,{C}_{i}(t)\le 1.\\ A\quad &{{{\rm{otherwise}}}}\end{array}\right.$$

14 $$\frac{d}{dt}{C}_{i}(t)=-\frac{{C}_{i}(t)}{{\tau }^{hom}}+{\left({z}_{i}^{ht}(t)\right)}^{2}$$

where *τ*^*hom*^ denotes a time constant, and ${z}_{i}^{ht}(t)$ denotes a synaptic trace that follows Equation (11) with its own time constant *τ*^*ht*^. Finally, excitatory weights were restricted to a range defined by lower and upper bounds ${w}_{exc}^{min}$ and ${w}_{exc}^{max}$, respectively. Nevertheless, excitatory weights never reached their upper bound in our simulations except in some protocols with blockage of neurons or plasticity mechanisms.

#### Inhibitory synaptic plasticity

Inhibitory synaptic plasticity took the form of a network activity-based STDP rule with an inhibitory synaptic weight *w*_*ij*_ from an inhibitory neuron *j* to an excitatory neuron *i* following^9^:

15 $$\frac{d}{dt}{w}_{ij}(t)={\eta }^{inh}G(t)\left[{z}_{j}(t){S}_{i}(t)+{z}_{i}(t){S}_{j}(t)+{S}_{j}(t)\right]$$

16 $$G(t)=H(t)-\gamma$$

17 $$\frac{d}{dt}H(t)=-\frac{H(t)}{{\tau }^{H}}+\mathop{\sum}\limits_{i\in exc}{S}_{i}(t)$$

where *η*^*inh*^ indicates a constant inhibitory learning rate, and *z*_*j*/*i*_ indicates either pre-synaptic or post-synapatic traces that follow Equation (11) with a shared time constant *τ*^*iSTDP*^. *G*(*t*) indicates a quantity that linearly depends on the difference between a hypothetical global secreted factor *H*(*t*) and the target local network activity level *γ*. *H*(*t*) indicates a low-pass-filtered version of the spikes fired by the population of excitatory neurons in the local network with time constant *τ*^*H*^. Note that when the network activity level falls below the target *γ*, *G*(*t*) < 0, and the inhibitory STDP rule (Equation (15)) becomes a unidirectional ‘depression-only’ rule^9^. However, when the network activity level raises above *γ*, the inhibitory rule becomes Hebbian. Thus, the primary goal of inhibitory synaptic plasticity was to control network activity and, thereby, support the stabilization of the overall network dynamics similarly to previous theoretical models^9,10,32^. In Extended Data Fig. 7, we also examined the ability of an alternative version of the inhibitory STDP rule in Equation (15) to support stable network dynamics:

18 $$\frac{d}{dt}{w}_{ij}(t)={\eta }^{inh}G(t)\left[{S}_{j}(t)\right]$$

where we retained the pre-synaptic-only term *S*_*j*_(*t*) and removed the Hebbian terms *z*_*j*_(*t*)*S*_*i*_(*t*) and *z*_*i*_(*t*)*S*_*j*_(*t*). In this alternative formulation of the inhibitory rule, *G*(*t*) and *H*(*t*) also follow Equations (16) and (17), respectively. Lastly, inhibitory weights were restricted to the range between ${w}_{inh}^{min}$ and ${w}_{inh}^{max}$, but they never reached their upper limit in our simulations except in some protocols with blockage of neurons or plasticity mechanisms.

### Network model

The model consisted of a stimulus population of *N*_*stim*_ = 4,096 Poisson neurons and a recurrent neural network corresponding to the hippocampus. Notably, the following anatomical evidence has motivated the use of a recurrent neural network to model the hippocampus: recurrent excitatory synapses in CA3 (ref. ^38^) and, to a less extent, in CA1 (ref. ^39^); feedforward synapses from DG to CA3 and from CA3 to CA1 (ref. ^40^); ‘backprojection’ synapses from CA1 to CA3 and DG^41^ and from CA3 to DG^42^; and recurrent inhibitory synapses in CA1 (ref. ^41^), CA3 (ref. ^38^) and DG^43^. The hippocampus network was composed of *N*_*exc*_ = 4,096 excitatory neurons and *N*_*inh*_ = 1,024 inhibitory neurons that were recurrently connected. Recurrent excitatory synapses onto excitatory neurons exhibited short-term and long-term excitatory synaptic plasticity, whereas excitatory synapses onto inhibitory neurons displayed only short-term plasticity. Inhibitory synapses onto inhibitory neurons were static, whereas inhibitory synapses onto excitatory neurons exhibited inhibitory synaptic plasticity. Feedforward excitatory synapses from the stimulus population to excitatory neurons in the hippocampus exhibited short-term and long-term excitatory synaptic plasticity. Recurrent synapses were randomly initialized following a uniform distribution, whereas feedforward synapses from the stimulus population had circular receptive fields centered at random locations (that is, each excitatory neuron in the hippocampus received projections from a small circular area in the stimulus population of radius *R*_*hpc*_ whose random center location followed a uniform distribution). Plasticity mechanisms were active in the entirety of all simulations except in protocols where plasticity was purposefully blocked. Recurrent synapses had a connection probability *ϵ*_*rec*_ and were initialized with specific weights (that is, *w*^*EE*^, *w*^*EI*^, *w*^*II*^ and *w*^*IE*^; E, excitatory, and I, inhibitory). Feedforward synapses had an initial weight *w*_*stim*_. For a complete list of network parameters, see Supplementary Table 2.

### Network simulation

Network simulations comprised multiple phases: burn-in, training, consolidation, probing and recall. Each simulation began with a brief burn-in period of duration *T*_*burn*_ that stabilized the activity of the hippocampus network under background input from the stimulus population at rate *ν*^*bg*^. Next, the training stimulus (Fig. 1c) was randomly presented to the hippocampus network in the training phase of duration *T*_*training*_ with stimulus-off and stimulus-on intervals both drawn from exponential distributions with means ${T}_{Off}^{\,training}$ and ${T}_{On}^{\,training}$, respectively. After the training phase, the training stimulus was randomly reactivated in the consolidation phase with stimulus-off and stimulus-on intervals also drawn from exponential distributions but with means ${T}_{Off}^{consolidation}$ and ${T}_{On}^{consolidation}$, respectively. This was motivated by recent experiments that showed that training-activated sensory neurons are selectively reactivated during post-training sleep and that this reactivation is essential for memory selectivity^11^. Hence, our model aimed to capture the role of sleep-dependent sensory reactivation in memory consolidation. At regular intervals throughout the consolidation phase (that is, consolidation time = 0, 1, …, *T*_*consolidation*_ hours), the network advanced to the probing phase when the training stimulus was randomly presented for a brief period *T*_*probing*_ with stimulus-off and stimulus-on intervals drawn from exponential distributions with means ${T}_{Off}^{probing}$ and ${T}_{On}^{probing}$, respectively. This allowed us to identify the set of engram cells that encode the training stimulus at any consolidation time point (see next subsection). We also subjected the network to the recall phase of duration *T*_*recall*_ after each consolidation interval (that is, consolidation time = 0, 1, …, *T*_*consolidation*_ hours). For each stimulus (that is, either the training stimulus or one of three novel stimuli; Fig. 1c), a separate recall session was conducted with partial cues of that individual stimulus being presented to the network. Hence, there was a total of four distinct parallel recall sessions (that is, one for the training stimulus and three for the novel stimuli) for each sampled consolidation interval. Cue-off and cue-on intervals also followed exponential distributions with means ${T}_{Off}^{recall}$ and ${T}_{On}^{recall}$, respectively. Each stimulus and partial cue is depicted in a 64 × 64 grid in Fig. 1c, and the overlap between the training stimulus and each novel stimulus is shown in Fig. 1d. During presentation or reactivation of a stimulus or cue, the stimulus population kept firing at the background rate *ν*^*bg*^, but the stimulus neurons that corresponded to a given stimulus or cue selectively increased their firing rate to *ν*^*stim*^. When blocking neurons (that is, stimulus, excitatory or inhibitory), their output and all their efferent synapses were blocked. For a complete list of simulation parameters, see Supplementary Table 2.

### Engram cells and recall metrics in simulations

In our simulations, engram cells were identified by computing the average stimulus-evoked firing rate of each neuron in the hippocampus network at different time points. First, a neuron was said to be an engram cell encoding the training stimulus at the end of the training phase if its average stimulus-evoked firing rate was above the threshold *ζ*^*thr*^ = 10 Hz for the last Δ*t*^*eng*^ = 60 s of the training phase. After training, we used the probing phase to identify engram cells after consolidation time had elapsed. Specifically, after consolidation time = 0, 1, …, *T*_*consolidation*_ hours, a neuron was regarded an engram cell encoding the training stimulus if its average stimulus-evoked firing rate was above the threshold *ζ*^*thr*^ = 10 Hz during the entire probing phase of duration *T*_*probing*_ = 60 s. Note that we used the last Δ*t*^*eng*^ = 60 s of the training phase to identify engram cells so that the time window used for engram cell identification in the training and probing phases had the same length in each case. Consequently, any differences between the ensembles of engram cells identified in the training and probing phases did not arise from differences in the time windows used for engram cell identification. In Extended Data Fig. 4j–o, we used an alternative, neuronal population-based approach to identify engram cells in the training and probing phases to examine the robustness of our modeling results. In this approach, we first computed the non-negative matrix of neuronal spike counts V (row = time bin, column = neuron) of the last Δ*t*^*eng*^ = 60 s of the training phase or the entire *T*_*probing*_ = 60 s of the probing phase using a time bin of 10 ms. Then, we used non-negative matrix factorization (NMF) to factorize V into two non-negative matrices W (that is, features matrix) and H (that is, coefficients matrix) such that V = WH. We set the number of features in the factorization (that is, number of columns in W) to 1 (that is, 1 engram ensemble encoding the training stimulus at any point in time), and a neuron was considered an engram cell encoding the training stimulus at a given time point if its coefficient supplied by H was above the threshold ${\zeta }_{NMF}^{thr}=0.5$. We used this neuronal population-based approach to identify engram cells because NMF is able to take into account the spike trains of the entire population of neurons, and it has an inherent clustering capability^44^ that has been previously used to detect cell assemblies in calcium imaging data^45^. Using either the single neuron-based (that is, average stimulus-evoked firing rate of individual neurons) or the neuronal population-based (that is, NMF of neuronal spike counts) approach to identify engram cells, a neuron may be an engram cell at one point and subsequently no longer be an engram cell at a later time point (that is, the neuron ‘dropped out of’ the engram^2^). Conversely, a neuron may not be an engram cell initially but later may become an engram cell (that is, the neuron ‘dropped into’ the engram^2^). In addition, a neuron may alternate between being and not being an engram cell over the course of consolidation. Naturally, a neuron may be an engram cell or a non-engram cell at the end of the training phase and remain so throughout the consolidation period. To examine the evolution of engrams with memory consolidation in our simulations, we measured the overlap between engram cells identified in the probing phase (that is, probing-activated or probing^+^ engram cells) and in the training phase (that is, training-activated or training^+^ engram cells) as a fraction of training^+^, probing^+^ or all neurons in the hippocampal network. We also tracked the size of the probing^+^ engram cell ensemble as a fraction of all neurons in the network as well as the hour-to-hour overlap between probing^+^ engram cell ensembles. Moreover, training stimulus neurons were considered stable sensory engram cells given the prominent role of training-activated sensory neurons in memory storage and consolidation^11^. Furthermore, an engram cell ensemble at a given time point was taken as activated upon presentation of a partial cue of either the training or a novel stimulus if its population firing rate (that is, average firing rate computed over all engram cells in the ensemble) was above the threshold *ζ*^*thr*^ = 10 Hz during presentation of the cue. We then measured recall as the number of instances when the engram cell ensemble was activated by a cue presentation divided by the total number of cue presentations in the recall phase. Moreover, we defined recall rate as the population firing rate of engram cells evoked by partial cues averaged across all cue presentations in the recall phase. We computed separate recall metrics for the training and novel stimuli given that cues of a single stimulus were presented in a given recall session (see previous subsection).

### Mice

C57BL/6J wild-type male mice were obtained from The Jackson Laboratory. Experiments using CCK-Cre mice employed the CCK-IRES-Cre knock-in line (stock no. 012706, The Jackson Laboratory). Experiments using PV-Cre mice employed the PV-IRES-Cre knock-in line (stock no. 017320, The Jackson Laboratory). Knock-in mice were maintained as hemizygotes. Mice had access to food and water ad libitum and were socially housed in numbers of 2–5 littermates until surgery. After surgery, mice were single housed. During housing, temperature was kept between 18 °C and 23 °C, and humidity was maintained between 40% and 60%. For behavioral experiments, all mice were male and 2–4 months old. All experiments were conducted in accordance with NIH guidelines and were approved by the Massachusetts Institute of Technology Department of Comparative Medicine and Committee on Animal Care.

### Mouse behavior

Experiments were conducted during the light cycle (7:00 to 19:00). Mice were randomly assigned to experimental groups for specific behavioral assays immediately after surgery. Mice were habituated to investigator handling for 1–2 min on three consecutive days. Handling took place in the holding room where the mice were housed. Before each handling session, mice were transported by wheeled cart to and from the vicinity of the behavior rooms to habituate them to the journey. All behavior experiments were analyzed blinded to experimental group. Following behavioral protocols, brain sections were prepared to confirm efficient viral labeling in hippocampal DG. Animals lacking adequate labeling were excluded before behavior quantification.

#### CFC

Two distinct contexts were employed. The conditioning context was a 29 × 25 × 22-cm chamber with grid floors and dim white lighting and scented with 0.25% benzaldehyde. The neutral context consisted of a 29 × 25 × 22-cm chamber with white perspex floors and red lighting and scented with 1% acetic acid. All mice were conditioned (120-s exploration, one 0.65-mA shock of 2-s duration at 120 s, 60-s post-shock period; second 0.65-mA shock of 2-s duration at 180 s, 60-s post-shock period; third 0.65-mA shock of 2-s duration at 240 s, 60-s post-shock period). After different intervals (1 h, 5 h, 12 h, 18 h and 24 h), mice were tested in the neutral context (3 min) followed by a recall test in the conditioning context (3 min) approximately 1 h later. Floors of chambers were cleaned with quatricide before and between runs. Mice were transported to and from the experimental room in their home cages using a wheeled cart. For experiments that included optogenetic manipulations (including Cal-Light active neuron labeling), the behavior chamber ceilings were customized to hold a rotary joint (Doric Lenses) connected to two 0.3-m optic fibers. All mice had optic fibers attached to their optic fiber implants before training and recall tests. Because optogenetic manipulations (that is, optic fibers) interfered with automated motion detection, freezing behavior was manually quantified for these experiments.

### Surgery

Animals were anesthetized with isoflurane for stereotaxic injections and were given 1 mg kg^−1^ meloxicam as analgesic before incisions. Virus was injected at 70 nl min^−1^ using a glass micropipette attached to a 10-ml Hamilton microsyringe. The needle was lowered to the target site and remained for 5 min before beginning the injection. After the injection, the needle stayed for 10 min before it was withdrawn. Coordinates for hippocampal DG were −2.0 mm AP, ±1.0 mm ML and −2.1 mm DV, and 250 nl of the virus was injected per hemisphere. For labeling and behavioral manipulation experiments using optogenetics, implants were lowered right above injection sites. The implant was secured to the skull with two jewelry screws, adhesive cement (C&B Metabond) and dental cement. Mice were given 1–2 mg kg^−^^1^ sustained-release buprenorphine as analgesic after surgeries and allowed to recover for at least 2 weeks before behavioral experiments.

### Activity-dependent labeling using Cal-Light

For Cal-Light experiments, a cocktail of three viruses was injected into hippocampal DG of wild-type mice, followed by bilateral optic fiber implants (200-μm core diameter, Newdoon). Viruses were AAV-TM-CaM-NES-TEV-N-AsLOV2-TEVseq-tTA (Addgene plasmid 92392), AAV-M13-TEV-C-P2A-tdTomato (Addgene plasmid 92391) and AAV-TRE-EGFP (Addgene plasmid 89875). These constructs were serotyped with AAV_5_ coat proteins and packaged by the Viral Core at Boston Children’s Hospital (∼4 × 10^12^ genome copies per milliliter (GC ml^−^^1^) viral titer). After recovery, active neurons during fear conditioning were tagged with EGFP by delivery of continuous blue light (473 nm, 15 mW at patch cord tip) for the entire duration of the training session (∼5 min). For *c-Fos* staining together with Cal-Light labeling, labeled animals were perfused 60 min after the specific behavioral epoch to capture endogenous *c-Fos* protein by immunohistochemistry (IHC). We used expression of the immediate early gene *c-Fos* as a marker of neuronal activity following standard practice in memory engram research^19,46,47^. Both Cal-Light labeling and *c-Fos* staining tag neurons that are highly activated during defined time windows without providing a quantitative measure of neuronal activity. For functional testing of Cal-Light-labeled neurons at different time points during memory consolidation, surgery mice were prepared using a cocktail of the three viruses in which the AAV_5_-TRE-EGFP was replaced by an AAV_5_-TRE-Chrimson-mCherry (Addgene plasmid 92207). Chrimson was activated during behavior with a 633-nm laser (10 mW, 20 Hz light).

### IHC

Mice were killed using an overdose of isoflurane and transcardially perfused with PBS, followed by 4% paraformaldehyde (PFA). Brains were extracted and incubated in 4% PFA at room temperature overnight. Brains were transferred to PBS, and 50-μm coronal slices were prepared using a vibratome. For immunostaining, each slice was placed in PBS + 0.2% Triton X-100 (PBS-T) with 5% normal goat serum for 1 h and then incubated with primary antibody at 4 °C for 16–24 h. Slices then underwent three wash steps for 10 min each in PBS-T, followed by a 2-h incubation with secondary antibody. After three more wash steps of 10 min each in PBS-T, slices were mounted on microscope slides. Antibodies used for staining were as follows: chicken anti-GFP (1:1,000, Life Technologies) and anti-chicken Alexa Fluor 488 (1:1,000), rabbit anti-c-Fos (1:500, Cell Signaling Technology) and anti-rabbit Alexa Fluor 555 (1:300), and nuclei were stained with DAPI (1:3,000, Sigma-Aldrich). For *c-Fos* staining in the CCK^+^ interneuron inhibition experiment, an anti-rabbit Alexa Fluor 633 (1:200) was used for the secondary antibody staining. Brain sections were imaged with a ×10 magnification objective on an Olympus fluorescence microscope.

### In situ hybridization

Mouse brain samples were carefully extracted, embedded in OCT compound (Tissue-Tek) and flash frozen. Coronal sections (16-μm thickness) were prepared on a cryostat (Leica) and stored at −80 °C until staining. ACD RNAScope multiplex fluorescent protocol was applied for mRNA fluorescence in situ hybridization (FISH) staining using fresh frozen tissues. In brief, charged slides with slices were fixed in pre-chilled 4% PFA for 30 min, followed by a series of dehydration steps using 50%, 70% and 100% ethanol. Sections were then permeabilized with ACD Protease IV for 30 min, followed by probe hybridization for 2 h at 40 °C. Fluorescent labeling of two probes per section was performed using four steps of Amp 1-FL to Amp 4-FL. Sections were stained with DAPI and stored at 4 °C. Mouse ACD probes for eYFP (cat. no. 312131), CCK (cat. no. 402271), Gad1 (cat. no. 400951), PV (cat. no. 421931) and c-Fos (cat. no. 316921) were used. Stained sections were imaged with a ×20 magnification objective on an Olympus confocal microscope.

### Cell counting

Images were processed using ImageJ software, and quantifications were performed manually from 3–5 sections per animal. All counting experiments were conducted blinded to experimental group. Researcher 1 trained the animals, prepared slices and randomized images, and Researcher 2 performed cell counting. Neuronal cell counts were normalized to the number of DAPI^+^ cells in the field of view.

### Ex vivo electrophysiological recordings

#### Brain slice preparation

CCK-Cre mice (7–9 weeks old) were injected in DG with a virus cocktail of Cal-Light (to label active neurons with TRE-EGFP), AAV_5_-Dlx5/6-DIO-hM4Di-mCherry (to inhibit inhibitory CCK^+^ interneurons using C21 during the cellular consolidation window) that we generated and AAV_5_-Dlx5/6-DIO-Chrimson-mCherry (to measure the strength of CCK^+^ connections to Cal-Light-labeled EGFP^+^ neurons in brain slices using 633-nm light) that we generated. About 12 h after CFC training, mice were anesthetized with isoflurane and decapitated, and their brains were quickly removed. Coronal slices (300-μm thickness) were prepared in oxygenated artificial cerebrospinal fluid (ACSF) solution at 4 °C using a Leica vibratome. ACSF contained (in mM): 125 NaCl, 3 KCl, 1.25 NaH_2_PO_4_, 2 MgSO_4_, 2 CaCl_2_, 25 NaHCO_3_ and 10 D-glucose, saturated with 95% O_2_/5% CO_2_ (pH 7.3, osmolarity of 300 mOsm). Slices were stored for 30 min at 33 °C (±0.5 °C) and then kept at room temperature until recordings.

#### Slice recordings

Electrophysiology data were collected using Clampex 10.7 software. Whole-cell recordings in voltage-clamp mode were performed using an IR-DIC microscope (Olympus) with a water immersion ×40 objective (NA = 0.8), equipped with four automatic manipulators (Luigs & Neumann) and a CCD camera (Hamamatsu). For all recordings, borosilicate glass pipettes were fabricated (Sutter Instrument) with resistances of 3.5–5 MΩ. IPSCs were pharmacologically isolated with 50 μM APV (Tocris) and 20 μM DNQX (Tocris) and recorded from cells that were voltage clamped at −70 mV using a high-Cl internal solution containing (in μM): 130 CsCl, 4 NaCl, 10 TEA, 10 HEPES, 2 Na_2_-ATP, 0.5 Na_3_-GTP and 0.2 EGTA. The osmolarity of this intracellular solution was 290 mOsm, and the pH was 7.25. Recordings were amplified using up to two dual-channel amplifiers (Molecular Devices), filtered at 2 kHz, digitized (20 kHz) and acquired through an ADC/DAC data acquisition unit (InstruTech) using custom software running on Igor Pro (WaveMetrics). Access resistance (Ra) was monitored throughout the duration of the experiment, and data acquisition was suspended whenever Ra was beyond 20 MΩ. To record oIPSCs, we used 2-ms 633-nm LED stimulations at 0.2, 0.5, 0.8, 1.1, 1.4 and 1.7 mW/mm^2^ generated through Polygon400 (Mightex) with built-in LED sources and delivered through the ×40 objective. Recordings were carried out on labeled DG granule cells (EGFP^+^) and unlabeled neighboring DG granule cells (EGFP^−^). In the experiments where CCK^+^ neurons were inhibited using C21 administration, oIPSCs were recorded using voltage-clamp mode at −70 mV with a high-Cl internal solution. Average peak responses were calculated from 10 sweeps, stimulated every 30 s with 0.8 mW/mm^2^ red light.

### In vivo calcium imaging

#### Experimental protocol

For calcium imaging experiments, wild-type mice that were 10–13 weeks old were used. Because these surgeries required the implantation of a GRIN lens targeting the upper blade of DG, we started by creating a guide track in the right hemisphere only. We then injected AAV_8_-Syn-jGCaMP7f virus (Addgene virus 104488-AAV8) in the right hemisphere (that is, these were unilateral injections). Ten minutes later, we implanted a 1.0-mm-diameter ProView Integrated Lens from Inscopix (part no. 1050-004637) targeting the right hemisphere DG, which was stabilized to the skull using 2–3 jewelry screws, adhesive cement (C&B Metabond) and dental cement. After 3.5 weeks for virus expression, mice were habituated to investigator handling and the attachment of a microendoscope that permits one-photon in vivo calcium imaging at single-cell resolution (nVista 3.0, Inscopix). To maximize animal movement while carrying the microendoscope, these animals were housed in a reverse light cycle room so that imaging experiments could be performed during their dark cycle (corresponding to 7:00 to 19:00 for the investigator). Individual animals were used for 5-min imaging sessions for a total of eight sessions across a 24-h period in the following order: home cage followed by contextual fear training; after a delay period of 1 h, home cage followed by neutral context followed by training context; and after a delay period of 23 h, home cage followed by neutral context followed by training context. Calcium events were captured at 20 Hz for one *z*-plane. Calcium imaging data were collected using Inscopix Acquisition Software 2.0.4. For image processing and single-cell identification analyses, we used Inscopix Data Processing Software 1.9.2. In brief, calcium imaging movies from each session were pre-processed using a spatial downsampling of 4 and a temporal downsampling of 2, which was followed by spatial filtering and motion correction. At this stage, the Δ*F*/*F* signal was calculated. Using the motion-corrected movie, we applied a constrained non-negative matrix factorization (CNMF) algorithm specifically for microendoscopic data (CNMF-E) to identify single DG cells. For each animal, we visually confirmed that every region of interest (ROI) identified by the CNMF-E approach indeed reflected only one cell. With the list of accepted ROIs/cells, using Inscopix Data Processing Software we performed image registration across the eight sessions per animal to be able to longitudinally track single cells across sessions. For the cells that could be tracked across sessions, calcium imaging traces were deconvolved and exported in a CSV file for downstream data and statistical analyses.

#### Longitudinal engram cell identification

To identify engram cells over time, we compared the average Δ*F*/*F* signal of each imaged cell in the training context and the home cage at different time points. Specifically, we first computed the average Δ*F*/*F* signal of each cell in each of the eight imaging sessions. We then computed a discrimination index (see subsection below) of the average Δ*F*/*F* signal of each cell in the training context and the home cage at three time points: for fear training, fear training versus home cage; for a delay = 1 h after fear training, recall in the training context versus home cage; and for a delay = 24 h after fear training, recall in the training context versus home cage. A cell was identified as an engram cell at a given time point if its Δ*F*/*F*-based discrimination index exceeded the threshold ${\zeta }_{disc}^{thr}=0.2$. We tracked the evolution of the engram by computing the overlap between engram cell ensembles identified at consecutive time points (that is, fear training, delay = 1 h and delay = 24 h) as a fraction of the engram cells identified in the previous time point, current time point or the number of imaged cells. In Extended Data Fig. 9a, we used an alternative, neuronal population-based approach to identify engram cells in our longitudinal calcium imaging experiments to evaluate the robustness of our findings. In this approach, we first took the matrix of Δ*F*/*F* calcium signals of the fear training session C_training_ (row = timestamp, column = neuron), and, for each entry, we subtracted the average Δ*F*/*F* of the corresponding neuron in the prior home cage session (that is, average of the corresponding column in the Δ*F*/*F* matrix of the prior home cage session C_home cage_). We then divided each entry in the resulting matrix by the sum of the average Δ*F*/*F* of the corresponding neuron in the prior home cage session and the average Δ*F*/*F* of the corresponding neuron in the current training session to arrive at the matrix C_normalized training_. Next, we subtracted the minimum value of C_normalized training_ from each of its entries to obtain the non-negative matrix ${{{{\rm{C}}}}}_{{{{\rm{normalized}}}}\,{{{\rm{training}}}}}^{{{{\rm{non}}}}-{{{\rm{negative}}}}}$. Subsequently, we employed an NMF-based approach to identify engram cells using ${{{{\rm{C}}}}}_{{{{\rm{normalized}}}}\,{{{\rm{training}}}}}^{{{{\rm{non}}}}-{{{\rm{negative}}}}}$ that was analogous to the NMF-based approach that we used to identify engram cells in our simulations (see previous subsection). Specifically, we used NMF to factorize ${{{{\rm{C}}}}}_{{{{\rm{normalized}}}}\,{{{\rm{training}}}}}^{{{{\rm{non}}}}-{{{\rm{negative}}}}}$ into two non-negative matrices W (that is, features matrix) and H (that is, coefficients matrix) such that ${{{{\rm{C}}}}}_{{{{\rm{normalized}}}}\,{{{\rm{training}}}}}^{{{{\rm{non}}}}-{{{\rm{negative}}}}}$ = WH. We set the number of features in the factorization (that is, number of columns in W) to 1 (that is, 1 engram ensemble encoding the fear memory at any point in time), and a neuron was considered an engram cell encoding the fear memory if its coefficient supplied by H was above the 99% quantile of the coefficients in H (that is, among the largest 1% of coefficients). To identify engram cells at delay = 1 h and 24 h, we repeated the procedure above replacing C_training_ with the Δ*F*/*F* matrix corresponding to recall in the training context at delay = 1 h and 24 h and replacing C_home cage_ with the Δ*F*/*F* matrix corresponding to the home cage session at delay = 1 h and 24 h. We used this neuronal population-based approach to identify engram cells because NMF is able to take into account the longitudinal calcium signals of the entire population of imaged neurons, and it has an inherent clustering property^44^ that has been used to identify cell assemblies in calcium imaging data^45^, as mentioned previously.

### Chemogenetic and optogenetic inhibition experiments

For chemogenetic (that is, hM4Di) neuronal activity manipulation experiments, we generated and injected an AAV_5_-Dlx5/6-DIO-hM4Di-mCherry virus (∼5.3 × 10^12^ GC ml^−1^ viral titer) into hippocampal DG of CCK-Cre mice. We used the second-generation DREADD agonist known as compound 21 (C21). This agonist was purchased in a water-soluble dihydrochloride form (Hello Bio). For each mouse, optimal chemogenetic activity was achieved using a target concentration of 2 mg kg^−1^ (injected intraperitoneally), which, in this study, was injected right after fear conditioning to inhibit DG CCK^+^ interneurons during the subsequent cellular consolidation window. Control mice were injected with a virus similar to the one used for the CCK^+^ inhibition group but lacking hM4Di. For optogenetic inhibition of CCK^+^ interneurons, we generated and injected an AAV_5_-Dlx5/6-DIO-eArch3.0-eYFP virus (∼3.8 × 10^12^ GC ml^−1^ viral titer) into hippocampal DG of CCK-Cre mice, followed by bilateral optic fiber implants targeting hippocampal DG. Control mice were injected with a virus similar to the one used for the CCK^+^ inhibition group but lacking eArch3.0. For optogenetic inhibition of PV^+^ interneurons, we injected an AAV_5_-EF1*α*-DIO-eArch3.0-eYFP virus (∼3 × 10^12^ GC ml^−1^ viral titer, UNC Vector Core) into hippocampal DG of PV-Cre mice, followed by bilateral optic fiber implants targeting hippocampal DG. Control mice were injected with a virus similar to the one used for the PV^+^ inhibition group but lacking eArch3.0. eArch3.0 was activated with a 570-nm laser (10 mW, constant light).

### Discrimination index

We defined a discrimination index between two quantities q_1_ and q_2_ as:

19 $${{{\rm{discrimination}}}}=\frac{{{{{\rm{q}}}}}_{1}-{{{{\rm{q}}}}}_{2}}{{{{{\rm{q}}}}}_{1}+{{{{\rm{q}}}}}_{2}}$$

We computed a discrimination index of memory recall in our simulations by setting q_1_ = recall_training stimulus_ and q_2_ = recall_novel stimulus_. recall_training stimulus_ and recall_novel stimulus_ denote recall levels when cues of the training stimulus and a novel stimulus are presented in the recall phase of our simulation protocol, respectively. Note that a separate discrimination index was calculated for each novel stimulus. We also computed a discrimination index of freezing behavior in our CFC experiments by setting q_1_ = freezing_training context_ and q_2_ = freezing_neutral context_. freezing_training context_ and freezing_neutral context_ denote freezing levels during memory recall in the training and neutral contexts, respectively. For both memory recall and freezing behavior, the discrimination index measured the ability of network models or mice to selectively recall the encoded memory only when cues of the training experience were presented.

In addition, we computed a discrimination index of the Δ*F*/*F* signal of each imaged cell in our calcium imaging experiment by setting q_1_ = $\Delta {{{F}}}/{{{{F}}}}_{{{{\rm{training}}}}\,{{{\rm{context}}}}}^{{{{\rm{cell}}}}}$ and q_2_ = $\Delta {{{F}}}/{{{{F}}}}_{{{{\rm{home}}}}\,{{{\rm{cage}}}}}^{{{{\rm{cell}}}}}$. $\Delta {{{F}}}/{{{{F}}}}_{{{{\rm{training}}}}\,{{{\rm{context}}}}}^{{{{\rm{cell}}}}}$ and $\Delta {{{F}}}/{{{{F}}}}_{{{{\rm{home}}}}\,{{{\rm{cage}}}}}^{{{{\rm{cell}}}}}$ denote the average Δ*F*/*F* signal of a given imaged cell in the training context and the home cage, respectively. This discrimination index was computed for each imaged cell at three time points (that is, fear training, delay = 1 h and delay = 24 h) to determine whether it was an engram cell in each instance (see subsection above). Lastly, we computed a discrimination index of the recall-evoked Δ*F*/*F* signal of each engram cell ensemble that we identified in our calcium imaging experiment by setting q_1_ = $\Delta {{{F}}}/{{{{F}}}}_{{{{\rm{training}}}}\,{{{\rm{context}}}}}^{{{{\rm{engram}}}}}$ and q_2_ = $\Delta {{{F}}}/{{{{F}}}}_{{{{\rm{neutral}}}}\,{{{\rm{context}}}}}^{{{{\rm{engram}}}}}$. $\Delta {{{F}}}/{{{{F}}}}_{{{{\rm{training}}}}\,{{{\rm{context}}}}}^{{{{\rm{engram}}}}}$ and $\Delta {{{F}}}/{{{{F}}}}_{{{{\rm{neutral}}}}\,{{{\rm{context}}}}}^{{{{\rm{engram}}}}}$ denote the average Δ*F*/*F* signal of an engram cell ensemble (that is, Δ*F*/*F* averaged across all cells in the ensemble) during recall in the training and neutral contexts, respectively. This discrimination index was computed at two time points (that is, delay = 1 h and 24 h), and it measured the ability of an engram cell ensemble to selectively respond only when mice were placed back in the training context.

### Statistics

Distributions of stimulus-evoked neuronal firing rates in simulations were compared using a two-sided Kolmogorov–Smirnov test. Distributions of Δ*F*/*F*-based discrimination indices of imaged cells in longitudinal calcium imaging experiments were compared using a two-sided Kolmogorov–Smirnov test. Distributions compared using a Kolmogorov–Smirnov test met required assumptions (that is, continuous variables). Freezing levels in CFC experiments were compared at multiple time points using a two-sided Wilcoxon signed-rank test between freezing in the training and neutral contexts. Discrimination indices between freezing levels in the training and neutral contexts in CFC experiments were compared against an average discrimination = 0 at multiple time points using a two-sided one-sample Wilcoxon signed-rank test. Memory recall at consolidation time = 24 h in the control simulation (Fig. 1i) and in the simulation with blockage of LTP during consolidation (Extended Data Fig. 4f) were compared using a two-sided Wilcoxon signed-rank test. Quantities compared using a Wilcoxon signed-rank test met required assumptions (that is, dependent samples, independence of paired observations, continuous dependent variable and ordinal level of measurement). For CFC experiments with Cal-Light active neuron labeling and *c-Fos* staining, a one-way ANOVA followed by Tukey’s honestly significant difference (HSD) post hoc test was used to compare ensemble overlap between neurons activated during both recall and training (*c-Fos*^+^ ∩ EGFP^+^) when memory recall was evaluated in the training context, neutral context or home cage. *c-Fos*^+^ ∩ EGFP^+^ as a fraction of training-activated neurons (EGFP^+^), recall-activated neurons (*c-Fos*^+^) and neuronal cell count (DAPI^+^) were each tested separately. Ensemble overlap data met required assumptions of one-way ANOVA (that is, independent samples, normality of residuals, homogeneity of variances and continuous dependent variable). For the experiments with optogenetic reactivation of Cal-Light-labeled neurons, we used an unpaired *t*-test to compare freezing levels in the neutral context between the control and the Cal-Light activation groups. Freezing level data compared using an unpaired *t*-test met required assumptions (that is, continuous variable, random sampling, homogeneity of variance and normality). For calcium imaging experiments, we computed Spearman’s rank correlation coefficient between the discrimination index of freezing levels and the discrimination index of recall-evoked Δ*F*/*F* signals of engram cell ensembles at delay = 1 h and 24 h (see subsection above). We also computed the associated *P* to test non-correlation. Discrimination data used to compute Spearman’s rank correlation met required assumptions (that is, paired observations and continuous variables). For electrophysiological recordings, a two-way repeated-measures ANOVA was used to compare oIPSC amplitudes between EGFP^+^ and EGFP^−^ neurons. oIPSC data met required assumptions of two-way repeated-measures ANOVA (that is, continuous dependent variable, normality of dependent variable, sphericity and absence of outliers). We used a paired *t*-test to compare normalized oIPSC amplitudes between EGFP^+^ and EGFP^−^ neurons. Normalized oIPSC data met required assumptions of paired *t*-test (that is, independent observations, normality of the differences between pairs and absence of outliers). For each test, the null hypothesis was rejected at the *P* < 0.05 significance level. No statistical methods were used to pre-determine sample sizes, but our sample sizes are similar to those reported in previous publications^3,4,19^.

### Simulation and data analysis details

In our simulations, we employed the forward Euler method to update neuronal state variables with a timestep Δ = 0.1 ms (with the exception of reference weights $\tilde{w}$ for which we set a longer timestep Δ_*long*_ = 1.2 s for efficiency reasons). Population activity (that is, average firing rate over all neurons in a given ensemble) was computed with a temporal resolution of 10 ms without smoothing or convolution. We computed 99% confidence intervals for mean metrics (that is, ensemble overlap, recall firing rate, recall and discrimination index) using a non-parametric bootstrap to provide an estimate of uncertainty and to facilitate their visualization.

### Code details

Simulation code was written in C++ employing the Auryn framework for spiking neural network simulation^48^. After several preliminary simulations, we found that setting the number of Message Passing Interface (MPI) ranks *N*_*ranks*_ = 16 minimized our simulation time with Auryn. Code used to analyze simulation output and experimental data was written in Python 3.11.

### Reporting summary

Further information on research design is available in the Nature Portfolio Reporting Summary linked to this article.

## Online content

Any methods, additional references, Nature Portfolio reporting summaries, source data, extended data, supplementary information, acknowledgements, peer review information; details of author contributions and competing interests; and statements of data and code availability are available at 10.1038/s41593-023-01551-w.

### Supplementary information

Supplementary Information Supplementary Discussion and Supplementary Table 2

Reporting Summary

Supplementary Table 1 Statistical tests in Figs. 3, 5 and 6

### Source data

Source Data Fig. 3 Statistical Source Data

Source Data Fig. 4 Statistical Source Data

Source Data Fig. 5 Statistical Source Data

Source Data Fig. 6 Statistical Source Data

Source Data Extended Data Fig. 6 Statistical Source Data

Source Data Extended Data Fig. 8 Statistical Source Data

## Data Availability

The data necessary to reproduce the simulations and data analyses reported in this study are available in a public repository^49^. Calcium imaging, individual mouse behavior and cell counting data are available in the Source Data files. Source data are provided with this paper.

## Code availability

The code used to perform the simulations and data analyses in this work is available in a public repository^49^.
