# Supplementary material for: Dynamic and selective engrams emerge with memory consolidation
Source: Nat Neurosci. 2024 Jan 19;27(3):561–72. doi: 10.1038/s41593-023-01551-w (PMC10917686; doi:10.1038/s41593-023-01551-w)
Supplement: Supplementary file 1 — Supplementary Discussion and Supplementary Table 2 [file 41593_2023_1551_MOESM1_ESM.pdf]

nature neuroscience

Article https://doi.org/10.1038/s41593-023-01551-w

Dynamic and selective engrams emerge with memory consolidation

In the format provided by the authors and unedited

Supplementary Information

Supplementary Discussion

We conducted our CFC experiments in the light cycle for mice, which could have had an effect on the evolution of the composition and selectivity of memory engrams. In particular, if our experiments had been performed in the dark cycle, we would expect that engram cell turnover and the emergence of memory selectivity would have been delayed relative to the light cycle case given that: I) neuronal reactivations in the hippocampus are mostly prevalent during NREM sleep,[1] and II) sleep-specific reactivation of training-activated sensory neurons is essential for the emergence of memory selectivity.[2] Despite a potential difference in timing between the light and dark cycles, we would expect that dynamic and selective engrams encode contextual fear memories in either case once neuronal reactivations have taken place.

The effects of blocking the reactivation of training-activated neurons have been investigated in previous experiments.[2,3] In particular, it has been shown that I) blocking the reactivation of training-activated place cells in the CA1 region of the hippocampus impaired spatial memory recall,[3] II) post-training sleep deprivation impaired visually-cued fear memory recall,[2] and III) blocking the reactivation of training-activated sensory neurons impaired visually-cued fear memory selectivity.[2] In our simulations, we specifically blocked the reactivation of training-activated sensory neurons and this disrupted memory selectivity in a manner consistent with previous experimental findings.[2] Notably, we did not explicitly model the effects of blocking the reactivation of training-activated hippocampal neurons, nor did we explicitly model the effects of post-training sleep deprivation and the consequent disruption of reactivation of training-activated neurons in multiple brain regions. However, either blocking the reactivation of training-activated neurons exclusively in the hippocampus or in multiple brain regions would disrupt long-term potentiation (LTP) of the efferent and afferent synapses of the blocked neurons given the known role of pre- and post-synaptic activity in the induction of LTP.[4] Importantly, we specifically blocked LTP during memory consolidation in our simulations and this impaired memory recall, in line with previous experimental findings where LTP in the hippocampus was optogenetically erased selectively during sleep.[5]

Evidence from various pharmacological manipulation studies suggests that memory encoding and consolidation are linked to moderate GABAergic transmission.[6] In particular, administering drugs that enhance GABA transmission either before or after fear training disrupts memory formation. Our work complements these observations by showing that inhibitory neurons become indispensable for memory selectivity via inhibitory synaptic plasticity during consolidation. Taken together, these findings suggest that inhibitory activity levels need to be maintained within a defined range to simultaneously enable the formation of memories and the emergence of selectivity. This is consistent with previous studies reporting that homeostatic inhibitory synaptic plasticity mechanisms can both control firing activity and support functional neuronal networks.[7–9] Notably, it has been shown that homeostatic synaptic scaling also contributes to the emergence of memory selectivity in conditioned taste aversion (CTA).[10] Specifically, blocking homeostatic synaptic scaling delayed the development of CTA memory selectivity. This suggests that synaptic scaling supports a gradual increase in memory selectivity by promoting competition among excitatory neurons as it scales down the strength of incoming synapses when neurons are highly active. However, CTA memory selectivity still eventually emerged even when synaptic scaling was blocked. This points to synaptic scaling promoting the emergence of memory selectivity by accelerating this process without being an essential homeostatic mechanism for the development of memory selectivity.

Previous work found that DG CCK$^+$ interneurons support memory selectivity by showing that inhibiting these interneurons during memory recall 24 or 48 h after contextual fear training impairs discrimination between the training context and a neutral context.[11] This study also showed that learning-induced inhibitory synaptic plasticity was correlated with the strength of fear learning. Our results expanded these findings in important ways. First, we showed that DG CCK$^+$ interneurons have a time-dependent role in mediating memory selectivity since inhibiting these interneurons impairs selectivity during recall 12 or 24 h but not 5 h after fear training. This was consistent with our experiments that showed that contextual fear memories are initially unselective but later become selective over the course of memory consolidation. Second, we found that inhibitory synaptic plasticity has a causal role in the development of memory selectivity by demonstrating that blocking the plasticity of DG CCK$^+$ efferent synapses prevented the emergence of engram selectivity. Importantly, we identified CCK$^+$ interneurons as mediators of memory selectivity but other interneuron types have also been implicated in the formation and evolution of engrams. For instance, PV$^+$ interneurons control the size of engrams in the lateral amygdala[12] whereas somatostatin-expressing (SST$^+$) interneurons constrain the size of hippocampal DG engrams.[13] Also, CA1 PV$^+$ interneuron

activity following encoding is required for memory consolidation[14] while PFC SST^+ interneuron activation and plasticity are critical for memory acquisition and expression.[15]

References

1. Ivan Skelin, Scott Kilianski, and Bruce L McNaughton. Hippocampal coupling with cortical and subcortical structures in the context of memory consolidation. *Neurobiology of learning and memory*, 160:21–31, 2019.

2. Brittany C Clawson, Emily J Pickup, Amy Ensing, Laura Geneseo, James Shaver, John Gonzalez-Amoretti, Meiling Zhao, A Kane York, Femke Roig Kuhn, Kevin Swift, et al. Causal role for sleep-dependent reactivation of learning-activated sensory ensembles for fear memory consolidation. *Nature Communications*, 12(1):1–13, 2021.

3. Igor Gridchyn, Philipp Schoenenberger, Joseph O'Neill, and Jozsef Csicsvari. Assembly-specific disruption of hippocampal replay leads to selective memory deficit. *Neuron*, 106(2):291–300, 2020.

4. Margarita Anisimova, Bas van Bommel, Rui Wang, Marina Mikhaylova, Jörn Simon Wiegert, Thomas G Oertner, and Christine E Gee. Spike-timing-dependent plasticity rewards synchrony rather than causality. *Cerebral Cortex*, 33(1):23–34, 02 2022.

5. Akihiro Goto, Ayaka Bota, Ken Miya, Jingbo Wang, Suzune Tsukamoto, Xinzhi Jiang, Daichi Hirai, Masanori Murayama, Tomoki Matsuda, Thomas J McHugh, et al. Stepwise synaptic plasticity events drive the early phase of memory consolidation. *Science*, 374(6569):857–863, 2021.

6. Steve R Makkar, Shirley Q Zhang, and Jacquelyn Cranney. Behavioral and neural analysis of GABA in the acquisition, consolidation, reconsolidation, and extinction of fear memory. *Neuropsychopharmacology*, 35(8):1625–1652, 2010.

7. Tim P Vogels, Henning Sprekeler, Friedemann Zenke, Claudia Clopath, and Wulfram Gerstner. Inhibitory plasticity balances excitation and inhibition in sensory pathways and memory networks. *Science*, 334(6062):1569–1573, 2011.

8. Friedemann Zenke, Everton J Agnes, and Wulfram Gerstner. Diverse synaptic plasticity mechanisms orchestrated to form and retrieve memories in spiking neural networks. *Nature Communications*, 6(1):1–13, 2015.

9. Ashok Litwin-Kumar and Brent Doiron. Formation and maintenance of neuronal assemblies through synaptic plasticity. *Nature Communications*, 5(1):1–12, 2014.

10. Chi-Hong Wu, Raul Ramos, Donald B Katz, and Gina G Turrigiano. Homeostatic synaptic scaling establishes the specificity of an associative memory. *Current Biology*, 31(11):2274–2285, 2021.

11. Xiaochen Sun, Max J Bernstein, Meizhen Meng, Siyuan Rao, Andreas T Sørensen, Li Yao, Xiaohui Zhang, Polina O Anikeeva, and Yingxi Lin. Functionally distinct neuronal ensembles within the memory engram. *Cell*, 181(2):410–423.e17, 2020.

12. Dano J Morrison, Asim J Rashid, Adelaide P Yiu, Chen Yan, Paul W Frankland, and Sheena A Josselyn. Parvalbumin interneurons constrain the size of the lateral amygdala engram. *Neurobiology of learning and memory*, 135:91–99, 2016.

13. Thomas Stefanelli, Cristina Bertollini, Christian Lüscher, Dominique Muller, and Pablo Mendez. Hippocampal somatostatin interneurons control the size of neuronal memory ensembles. *Neuron*, 89(5):1074–1085, 2016.

14. Nicolette Ognjanovski, Samantha Schaeffer, Jiaxing Wu, Sima Mofakham, Daniel Maruyama, Michal Zochowski, and Sara J Aton. Parvalbumin-expressing interneurons coordinate hippocampal network dynamics required for memory consolidation. *Nature Communications*, 8(1):1–14, 2017.

15. Kirstie A Cummings and Roger L Clem. Prefrontal somatostatin interneurons encode fear memory. *Nature Neuroscience*, 23(1):61–74, 2020.

Supplementary Table 2. List of network simulation parameters. Value source indicated as below:
(a) for values taken from Friedemann Zenke, Everton J Agnes, and Wulfram Gerstner. Diverse synaptic plasticity mechanisms orchestrated to form and retrieve memories in spiking neural networks. *Nature Communications*, 6(1):1–13, 2015.
(b) for values chosen somewhat arbitrarily without targeted optimization.
(c) for values optimized over several preliminary simulations.

**Neural Populations**		
**Parameter**	**Value[source]**	**Description**
N_{exc}	4096[a]	Size of population of excitatory neurons in the hippocampus
N_{inh}	1024[a]	Size of population of inhibitory neurons in the hippocampus
N_{stim}	4096[a]	Size of population of stimulus neurons
**Network Connectivity**		
**Parameter**	**Value[source]**	**Description**
ϵ_{rec}	0.05[a]	Probability of connection of recurrent synapses
R_{hpc}	8[a]	Radius of receptive field of excitatory neurons
w^{EE}	0.1[a]	Initial weight of recurrent excitatory synapses onto excitatory neurons
w^{EI}	0.6[a]	Fixed weight of recurrent excitatory synapses onto inhibitory neurons
w^{II}	0.2[a]	Fixed weight of recurrent inhibitory synapses onto inhibitory neurons
w^{IE}	0.2[a]	Initial weight of recurrent inhibitory synapses onto excitatory neurons
w_{stim}	0.5[a]	Initial weight of feedforward excitatory synapses onto excitatory neurons
**Neuron Model**		
**Parameter**	**Value[source]**	**Description**
τ^{m}	20 ms[a]	Membrane time constant
U^{rest}	-70 mV[a]	Membrane resting potential
U^{exc}	0 mV[a]	Excitatory reversal potential
U^{inh}	-80 mV[a]	Inhibitory reversal potential
τ^{thr}	5 ms[a]	Threshold time constant
ϑ^{rest}	-50 mV[a]	Threshold resting value
ϑ^{spike}	100 mV[a]	Threshold value immediately after spike
**Synapse Model**		
**Parameter**	**Value[source]**	**Description**
τ^{gaba}	10 ms[a]	GABA decay time constant
τ^{a}	100 ms[a]	Adaptation time constant
Δ^{a}	0.1[a]	Adaptation strength
α^{E}	0.2[a]	AMPA/NMDA ratio for excitatory neurons
α^{I}	0.3[a]	AMPA/NMDA ratio for inhibitory neurons
τ^{ampa}	5 ms[a]	AMPA decay time constant
τ^{nmda}	100 ms[a]	NMDA decay time constant
**Short-Term Plasticity Model**		
**Parameter**	**Value[source]**	**Description**
τ^{d}_{EE}	150 ms[a]	Depression time constant for excitatory synapses onto excitatory neurons
τ^{d}_{EI}	200 ms[a]	Depression time constant for excitatory synapses onto inhibitory neurons
τ^{f}	600 ms[a]	Facilitation time constant for excitatory synapses
U	0.2[a]	Initial release probability for excitatory synapses

Long-Term Excitatory Synaptic Plasticity Model		
**Parameter**	**Value$^{(source)}$**	**Description**
η^{exc}	1 x $10^{-3(a)}$	Learning rate of excitatory synapses
λ^{β}	$50^{(a)}$	β/η^{exc} ratio
τ^{cons}	20 min$^{(a)}$	Synaptic consolidation time constant
λ^{δ}	$0.02^{(a)}$	δ/η^{exc} ratio
A	$1^{(a)}$	LTP rate
τ^{+}	20 ms$^{(a)}$	Time constant of presynaptic trace for excitatory synaptic plasticity
τ^{-}	20 ms$^{(a)}$	Time constant of postsynaptic trace for excitatory synaptic plasticity
τ^{slow}	100 ms$^{(a)}$	Time constant of slow postsynaptic trace for excitatory synaptic plasticity
P	$20^{(a)}$	Potential strength
w^{P}	$0.5^{(a)}$	Upper fixed point of reference weight potential
$\tilde{w}$	$0.0^{(a)}$	Initial reference weight
τ^{hom}	10 min$^{(a)}$	Time constant of homeostatic regulation
τ^{ht}	100 ms$^{(a)}$	Time constant of postsynaptic trace for homeostatic regulation
w_{exc}^{max}	$5.0^{(a)}$	Maximum excitatory synaptic weight
w_{exc}^{min}	$0.0^{(a)}$	Minimum excitatory synaptic weight
**Inhibitory Synaptic Plasticity Model**		
**Parameter**	**Value$^{(source)}$**	**Description**
λ^{η}	$50^{(a)}$	η^{exc}/η^{inh} ratio
γ	4 Hz$^{(a)}$	Target activity level for excitatory neurons in the hippocampus
τ^{iSTDP}	20 ms$^{(a)}$	Time constant of pre- and postsynaptic traces for inhibitory synaptic plasticity
τ^{H}	10 s$^{(a)}$	Time constant of global secreted factor
w_{inh}^{max}	$5.0^{(a)}$	Maximum inhibitory synaptic weight
w_{inh}^{min}	$0.0^{(a)}$	Minimum inhibitory synaptic weight
**Stimulus Model**		
**Parameter**	**Value$^{(source)}$**	**Description**
ν^{bg}	5 Hz$^{(a)}$	Background firing rate of stimulus population
ν^{stim}	15 Hz$^{(c)}$	Firing rate of neurons matching given stimulus when that stimulus is activated
$T_{On}^{training}$	1 s$^{(c)}$	Mean stimulus-on period in the training phase
$T_{Off}^{training}$	2 s$^{(c)}$	Mean stimulus-off period in the training phase
$T_{On}^{probing}$	1 s$^{(c)}$	Mean stimulus-on period in the probing phase
$T_{Off}^{probing}$	2 s$^{(c)}$	Mean stimulus-off period in the probing phase
$T_{On}^{consolidation}$	1 s$^{(c)}$	Mean stimulus-on period in the consolidation phase
$T_{Off}^{consolidation}$	3 s$^{(c)}$	Mean stimulus-off period in the consolidation phase
T_{On}^{recall}	1 s$^{(c)}$	Mean stimulus-on period in the recall phase
T_{Off}^{recall}	2 s$^{(c)}$	Mean stimulus-off period in the recall phase
**Network Simulation**		
**Parameter**	**Value$^{(source)}$**	**Description**
T_{burn}	120 s$^{(b)}$	Duration of burn-in period prior to training
$T_{training}$	5 min$^{(c)}$	Duration of training phase
$T_{consolidation}$	24 h$^{(b)}$	Duration of consolidation phase
$T_{probing}$	60 s$^{(c)}$	Duration of probing phase
T_{recall}	90 s$^{(b)}$	Duration of recall phase
Δ	0.1 ms$^{(a)}$	Time step for updating neuronal state variables except for reference weights $\tilde{w}$
Δ_{long}	1.2 s$^{(a)}$	Time step for updating reference weights $\tilde{w}$
N_{ranks}	$16^{(c)}$	Number of MPI ranks in Auryn simulations
