# Supplementary material for: Dynamic and selective engrams emerge with memory consolidation
Source: Nat Neurosci. 2024 Jan 19;27(3):561–72. doi: 10.1038/s41593-023-01551-w (PMC10917686; doi:10.1038/s41593-023-01551-w)
Supplement: Supplementary file 2 — Reporting Summary [file 41593_2023_1551_MOESM2_ESM.pdf]

nature portfolio

Corresponding author(s): Claudia Clopath

Last updated by author(s): Dec 2, 2023

Reporting Summary

Nature Portfolio wishes to improve the reproducibility of the work that we publish. This form provides structure for consistency and transparency in reporting. For further information on Nature Portfolio policies, see our Editorial Policies and the Editorial Policy Checklist.

Statistics

For all statistical analyses, confirm that the following items are present in the figure legend, table legend, main text, or Methods section.

n/a	Confirmed	
☐	☒	The exact sample size (n) for each experimental group/condition, given as a discrete number and unit of measurement
☐	☒	A statement on whether measurements were taken from distinct samples or whether the same sample was measured repeatedly
☐	☒	The statistical test(s) used AND whether they are one- or two-sided<br>*Only common tests should be described solely by name; describe more complex techniques in the Methods section.*
☒	☐	A description of all covariates tested
☐	☒	A description of any assumptions or corrections, such as tests of normality and adjustment for multiple comparisons
☐	☒	A full description of the statistical parameters including central tendency (e.g. means) or other basic estimates (e.g. regression coefficient) AND variation (e.g. standard deviation) or associated estimates of uncertainty (e.g. confidence intervals)
☐	☒	For null hypothesis testing, the test statistic (e.g. F, t, r) with confidence intervals, effect sizes, degrees of freedom and P value noted<br>*Give P values as exact values whenever suitable.*
☒	☐	For Bayesian analysis, information on the choice of priors and Markov chain Monte Carlo settings
☒	☐	For hierarchical and complex designs, identification of the appropriate level for tests and full reporting of outcomes
☐	☒	Estimates of effect sizes (e.g. Cohen's d, Pearson's r), indicating how they were calculated

Our web collection on statistics for biologists contains articles on many of the points above.

Software and code

Policy information about availability of computer code

Data collection	We wrote C++ code using the Auryn framework for spiking neural network simulation version 6928b97 to perform all reported simulations. The code was deposited in a public repository at https://zenodo.org/doi/10.5281/zenodo.10251086. Ex vivo electrophysiology data were collected using the Clampex 10.7 software and in vivo calcium imaging data were collected using the Inscopix Acquisition Software 2.0.4.
Data analysis	We wrote Python 3.11 code to analyze the results of all reported simulations and experiments. The code was deposited in a public repository at https://zenodo.org/doi/10.5281/zenodo.10251086. Following acquisition, calcium imaging data was processed using the Inscopix Data Processing Software 1.9.2.

For manuscripts utilizing custom algorithms or software that are central to the research but not yet described in published literature, software must be made available to editors and reviewers. We strongly encourage code deposition in a community repository (e.g. GitHub). See the Nature Portfolio guidelines for submitting code & software for further information.

Data

Policy information about availability of data

All manuscripts must include a data availability statement. This statement should provide the following information, where applicable:

- Accession codes, unique identifiers, or web links for publicly available datasets
- A description of any restrictions on data availability
- For clinical datasets or third party data, please ensure that the statement adheres to our policy

The data necessary to reproduce the simulations and data anlyses reported in this study are available in a public repository at https://zenodo.org/doi/10.5281/zenodo.10251086. Calcium imaging, individual mouse behavior, and cell counting data are available in the Source Data files.

Field-specific reporting

Please select the one below that is the best fit for your research. If you are not sure, read the appropriate sections before making your selection.

☒ Life sciences ☐ Behavioural & social sciences ☐ Ecological, evolutionary & environmental sciences

For a reference copy of the document with all sections, see nature.com/documents/nr-reporting-summary-flat.pdf

Life sciences study design

All studies must disclose on these points even when the disclosure is negative.

Sample size	No statistical methods were used to pre-determine sample sizes but our sample sizes are similar to those reported in previous publications (https://doi.org/10.1038/s41593-018-0318-7, https://doi.org/10.1038/nature11028, https://doi.org/10.1126/science.1164139). The selected sample sizes were sufficient given the reproducibility of our results across trials.
Data exclusions	No data were excluded from the computational analyses. For mouse experiments, if the viral targeting missed the brain region of interest, animals were removed prior to data analysis.
Replication	The simulations reported in the manuscript were performed on three different machines and the main findings of the study were replicated each time. Mouse experiments, including behavior, cell counting, and physiology, were performed in at least two independent batches yielding consistent results.
Randomization	All spiking neural networks simulated in the study were randomly initialized (with random seeds) before training. Mice were randomly assigned to experimental groups for specific behavioral assays immediately after surgery.
Blinding	Blinding was not performed for the analysis of simulation results since simulation output was automatically processed by our custom data analysis code without human intervention. All cell counting experiments were conducted blind to experimental group: Researcher 1 trained the animals, prepared slices, and randomized images, while Researcher 2 performed cell counting. Similarly, mouse behavior and slice electrophysiology experiments were conducted blind to experimental group information.

Reporting for specific materials, systems and methods

We require information from authors about some types of materials, experimental systems and methods used in many studies. Here, indicate whether each material, system or method listed is relevant to your study. If you are not sure if a list item applies to your research, read the appropriate section before selecting a response.

Materials & experimental systems

n/a	Involved in the study	
☐	☒	Antibodies
☒	☐	Eukaryotic cell lines
☒	☐	Palaeontology and archaeology
☐	☒	Animals and other organisms
☒	☐	Human research participants
☒	☐	Clinical data
☒	☐	Dual use research of concern

Methods

n/a	Involved in the study	
☒	☐	ChIP-seq
☒	☐	Flow cytometry
☒	☐	MRI-based neuroimaging

Antibodies

Antibodies used	Chicken anti-GFP (1:1000, Life Technologies, Catalog # A10262), anti-chicken Alexa-488 (1:1000, Life Technologies, Catalog # A-11039), rabbit anti-c-Fos (1:500, Cell Signaling Technology, Catalog # 2250, Clone Name 9F6), anti-rabbit Alexa-555 (1:300, Life Technologies, Catalog # 21428), anti-rabbit Alexa-633 (1:200, Life Technologies, Catalog # 21070).
Validation	All these antibodies have been validated by the manufacturer and other researchers using cell lines, western blots, and mouse brain tissue.

Animals and other organisms

Policy information about studies involving animals; ARRIVE guidelines recommended for reporting animal research

Laboratory animals	C57BL/6J wild type male mice (Jackson Laboratory), CCK-IRES-Cre knock-in mice (Stock No. 012706, Jackson Laboratory), and PV-IRES-Cre knock-in mice (Stock No. 017320, Jackson Laboratory). All mouse lines were maintained as hemizygotes. For behavioral experiments, all mice were male and 2-4 months old.

Wild animals	The study did not involve animals captured in the field.
Field-collected samples	The study did not involve samples collected from the field.
Ethics oversight	All experiments were conducted in accordance with U.S. National Institutes of Health (NIH) guidelines and were approved by the Massachusetts Institute of Technology Department of Comparative Medicine and Committee on Animal Care.

Note that full information on the approval of the study protocol must also be provided in the manuscript.
